# Forward Osmosis Application in Manufacturing Industries: A Short Review

**DOI:** 10.3390/membranes8030047

**Published:** 2018-07-23

**Authors:** Anita Haupt, André Lerch

**Affiliations:** Process Engineering in Hydro Systems, Institute of Urban and Industrial Water Management, Technische Universität Dresden, 01062 Dresden, Germany; andre.lerch@tu-dresden.de

**Keywords:** forward osmosis, direct osmosis, manufacturing industry, lab-scale set-up, industrial wastewater

## Abstract

Forward osmosis (FO) is a membrane technology that uses the osmotic pressure difference to treat two fluids at a time giving the opportunity for an energy-efficient water and wastewater treatment. Various applications are possible; one of them is the application in industrial water management. In this review paper, the basic principle of FO is explained and the state-of-the-art regarding FO application in manufacturing industries is described. Examples of FO application were found for food and beverage industry, chemical industry, pharmaceutical industry, coal processing, micro algae cultivation, textile industry, pulp and paper industry, electronic industry, and car manufacturing. FO publications were also found about heavy metal elimination and cooling water treatment. However, so far FO was applied in lab-scale experiments only. The up-scaling on pilot- or full-scale will be the essential next step. Long-term fouling behavior, membrane cleaning methods, and operation procedures are essential points that need to be further investigated. Moreover, energetic and economic evaluations need to be performed before full-scale FO can be implemented in industries.

## 1. Introduction

### 1.1. Demand for Innovative, Energy-Efficient Water and Wastewater Treatment

Many of the sustainable development goals (SDG), provided by the United Nations in 2015, are related to a sufficient water supply [1,2]. Agriculture consumes 70% of the world’s freshwater, followed by industry that consumes 19% [3]. For this reason, efficient water usage in agriculture but also in industrial production processes is necessary to achieve all SDG. Industries nowadays apply treatment technologies to treat water and wastewater. Often, recycling of water is accomplished and freshwater demand as well as wastewater amounts are reduced. However, most treatment technologies consume large amounts of energy [4]. Aiming for sustainability, the energy efficiency of water and wastewater treatment needs to be improved.

Membrane filtration processes are often used in water recycling processes. Conventional membrane technologies are micro-, ultra-, and nano-filtration as well as reverse osmosis (MF, UF, NF, RO). They use a transmembrane pressure difference which is generated by pumping. Thus, water molecules pass through the membrane and impurities are rejected. The energy demand of these pressure-driven membrane processes is very high. In contrast to that, forward osmosis (FO) is a membrane technology that uses the osmotic pressure difference between two solutions to generate a water flow through the membrane [4]. Therefore, only little external energy is required and energy-efficient water treatment is achieved [5]. Membrane filtration processes are often applied off-line or off-site in relation to the regular production processes. More beneficial, however, is the application in-situ or in-line enabling direct recovery and recycle of resources or water. Several examples in various fields, including forward osmosis, have been published [6,7,8,9].

For these reasons, forward osmosis is potentially applicable in industrial water treatment to enhance energy efficiency. It might even be applied for wastewaters that so far cannot be treated by pressure-driven membrane technologies [10]. Since there is a large variety of industrial wastewaters, FO might also be suitable to treat two wastewaters in only one treatment step providing one concentrated wastewater and one diluted wastewater. This energy-efficient combination could lead to an optimized, more economic water and resources management in industries.

In order to show the current state of the art, a literature review was performed about forward osmosis application in manufacturing industries. The results of which are presented in this paper. There are numerous other literature reviews about forward osmosis in general [4,10,11,12,13,14,15,16,17] or special aspects, e.g., membrane fouling [18,19,20], membrane characteristics [21], draw solutions [22,23,24], hybrid processes [25,26], application in seawater desalination [27,28,29,30,31], application in wastewater treatment [32], application in produced water treatment [33,34], application in food processing [35], application for resource recovery from municipal wastewater [36], and osmotic membrane bioreactors [37].

### 1.2. Forward Osmosis Technology

**Figure 1:**
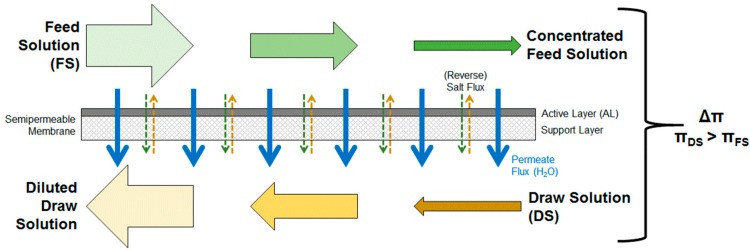
Schematic illustration of the forward osmosis process with membrane active layer facing towards the feed solution (ALFS or FO-mode).

Forward osmosis is a technology that uses a membrane to treat two liquid streams. Figure 1 illustrates the operating principle. On one side of the membrane is the so-called feed solution (FS). The FS has a low osmotic pressure. On the other side of the membrane is the so-called draw solution (DS) that has a higher osmotic pressure. A semi-permeable membrane separates FS and DS. Due to the difference in osmotic pressure, water passes through the membrane from the FS to the DS side. This diffusing water dilutes the DS; simultaneously, the FS is concentrated. Usually, no physical pressure is needed. Therefore, the only energy demand results from the pumping of FS and DS through the flow channels next to the membrane. Compared to other treatment technologies, FO offers the following advantages:

- low energy consumption,
- simultaneous treatment of two streams in one treatment step,
- easy removability of fouling layers due to absence of compression,
- treatment of liquids that are not suitable for other membrane processes.

An important aspect in FO operation is the concentration polarization (CP) and its influence on the water passing through the membrane (permeate flux) [38]. Concentration polarization describes the fact that due to the water flux through the membrane FS is concentrated on the membrane surface. This phenomena also occurs in conventional membrane filtration processes such as RO. In FO it is called concentrative CP. The difference in FO is that on the other side of the membrane, DS is diluted and so-called dilutive CP takes place. Due to CP, the real effective osmotic pressure gradient is lower than the osmotic pressure difference between inlet FS and DS. Thus, permeate fluxes are lower than expected. Concentration polarization occurs on the membrane surface (external concentration polarization ECP) and within the porous support layer of the membrane (internal concentration polarization ICP).

FO membranes usually consist of an active layer (AL) and a porous support layer (SL). The membrane can either be used with active layer facing the FS (ALFS or FO mode) or with active layer facing the DS (ALDS or PRO mode). In ALDS mode, concentration polarization is less severe and permeate fluxes are higher [38]. However, in many cases the ALFS mode is used because fouling can be removed easier from the dense active layer than from the porous support layer [38].

An effect occurring during FO treatment is the so-called reverse salt flux. This term describes that substances from the DS diffuse into the FS through the membrane due to concentration differences, possibly changing FS composition [9].

**Figure 2:**
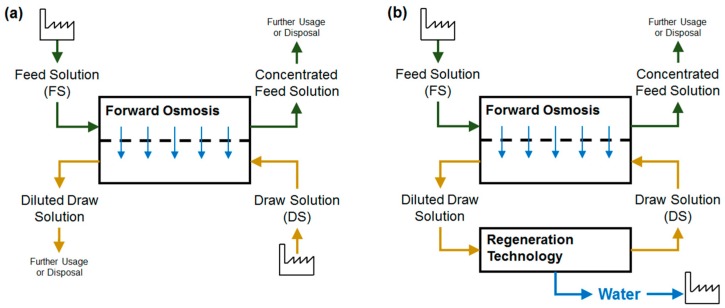
FO process with direct usage of FS and DS (**a**); FO process with DS regeneration step (**b**).

In FO process, the FS is concentrated and the DS is diluted. In some cases, two liquid streams might be combined where these effects are desired. Then, no additional technology is needed. In many cases however, an artificial DS is used and recycled. For this, the DS has to be concentrated after FO and a regeneration step is required (Figure 2) [7,8]. Pure water is obtained as a valuable product. Those combined technologies are often referred to as “FO hybrid technologies”. In contrast, the term “direct FO application” is used in this review paper to describe cases where no DS regeneration is necessary.

Potential regeneration steps are all technologies that somehow recover water from a solution. They are evaporation, heating, membrane distillation, and pressurized membrane technologies (e.g., RO) [26]. Furthermore, there are studies where the DS substance is extracted from the diluted DS, e.g., by magnetic field or electric current application [22]. The DS regeneration strongly depends on the used DS. Since additional treatment technologies require energy, the overall energy demand of hybrid FO processes has to be taken into consideration. In one example for seawater desalination, the specific energy demand is 2.5–4.0 and 1.3–1.5 with conventional RO and FO with low pressure RO, respectively [39]. Mazlan et al. stated that for seawater desalination with 75% water recovery the energy demand is 2.3 kWh/m^3^ using two-stage RO; applying FO with different DS types and regeneration technologies it is between 1.2 and 3.3 kWh/m^3^ [5]. FO alone requires less than 0.25 kWh/m^3^ energy [40]. For this reason, an energy-efficient regeneration of a suitable DS is crucial for FO hybrid processes.

The high osmotic pressure of the DS is usually achieved through high concentrations of solutes. In some applications, combined treatment of two (waste) water streams might be possible. Here, no synthetic DS would be necessary. However, in other applications, only one water stream is treated by FO and an artificial DS is necessary.

Johnson et al. summarize the current state of knowledge about synthetic DS [22]. A suitable DS has to be chosen considering osmotic pressure, viscosity, reverse salt flux, internal concentrative polarization, availability, costs, regeneration, and toxicity. Potential synthetic DS are:

- gases and volatile compounds,
- inorganic draw solutes (e.g., salts),
- organic draw solutes (e.g., sugar, organic ionic liquids, switchable polarity solvents (SPS), organic ionic salts, polyelectrolytes, polymers, hydrogels),
- functionalized nanoparticles.

**Figure 3:**
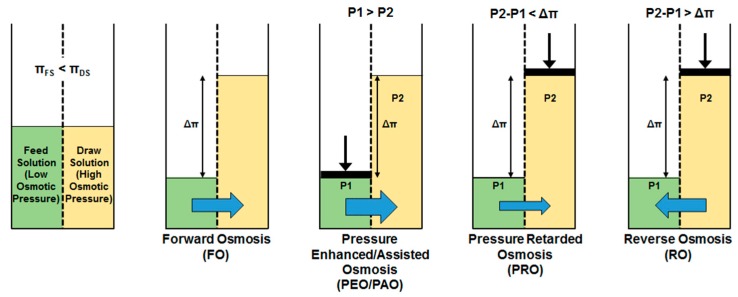
Osmotic membrane technologies (reprinted from [41] with permission from author).

Besides the “normal” forward osmosis process, there are similar FO-related processes that somehow utilize physical pressure. They are pressure-assisted or pressure-enhanced osmosis (PAO or PEO), and pressure-retarded osmosis (PRO) (Figure 3).

In the 1970s, first FO experiments used RO membranes [42,43]. However, FO permeate fluxes were very low because of the high concentration polarization and no more efforts were made to establish forward osmosis as water treatment technology. This is because RO membranes usually have two layers: a dense active layer and a porous support layer [44]. The support layer requires a certain thickness due to the necessity to withstand high physical pressures. Due to the thick support layer, internal CP is very high in FO application producing only low permeate fluxes.

**Figure 4:**
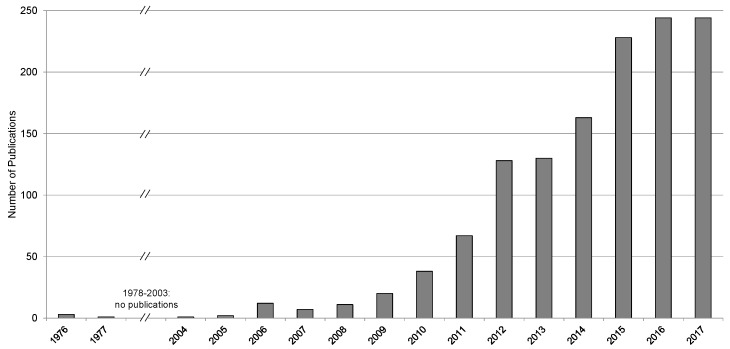
Number of annual publications on forward osmosis (Database: Google Scholar; searching exact phrase “forward osmosis” in the title of the article; patents and citations excluded).

In the course of ongoing membrane development, special FO membranes were developed with a thinner support layer [21,45,46]. As no high hydraulic pressure occurs in FO, the support layer does not need to be as thick as in RO. With a thinner support layer, concentration polarization decreases allowing higher permeate fluxes. Since 2004, the number of scientific publications on forward osmosis has increased rapidly (Figure 4). This shows that FO is of high interest having the potential to treat water and wastewater efficiently. Still, the wastewater that originates from membrane manufacturing itself has to be taken into account when considering overall process sustainability [47].

## 2. Forward Osmosis Application—State of Implementation

### 2.1. Bench- and Lab-Scale

**Figure 5:**
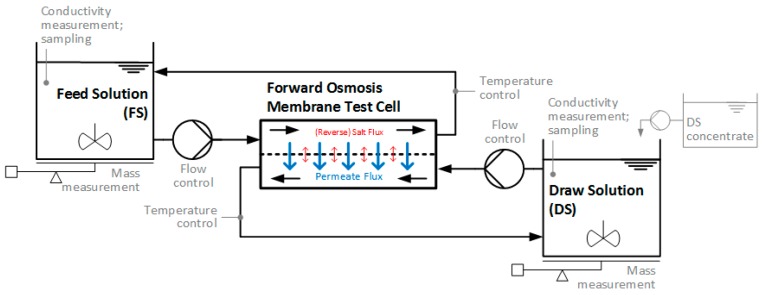
Typical lab-scale forward osmosis set-up (optional parts in grey).

Reported bench- or lab-scale FO set-ups are usually very similar (Figure 5). They include a membrane test cell with a FS and DS circulation loop driven by pumps. The membrane test cells mostly include a flat sheet membrane sample and a flow channel on both sides of the membrane. Spacers are sometimes used in the flow channels. The membrane active layer can be placed either towards the FS (ALFS or FO mode) or towards the DS (ALDS or PRO mode). FS and DS flow velocity is usually adjusted via the circulation pumps and flow rate measurement. FS and DS can flow concurrently or counter-currently in the flow channels, resulting in different development of osmotic pressure difference along the membrane. Theoretically, membrane test cells could also be constructed to enable cross-current flow. However, to the authors’ knowledge no such test cell has been used in lab-scale experiments for industrial wastewater application so far.

FS and DS are stored in containers of varying sizes and pumped through the test cell back into the containers, the contents of which are usually stirred. In the course of FO experiment, water permeates from the FS into the DS. Thus, using this type of batch experiment, FS is concentrated and DS is diluted and the osmotic pressure difference across the membrane decreases. In some cases, DS concentration is kept constant by dosing DS concentrate into the DS container, either continuously or periodically. Often DS conductivity measurements control this dosage. This way, only the FS osmotic pressure increases and the osmotic pressure difference between FS and DS declines more slowly. Occasionally, water baths or other devices are used to control the FS and DS temperature.

FS or DS mass measurements deliver data for permeate flux calculation. Conductivity measurements or sample analyzes provide information about FS and DS composition and substance fluxes through the membrane (e.g., reverse salt flux). Since membrane test cells often consist of acrylic glass, the membrane surface can be monitored during the experiments.

**Figure 6:**
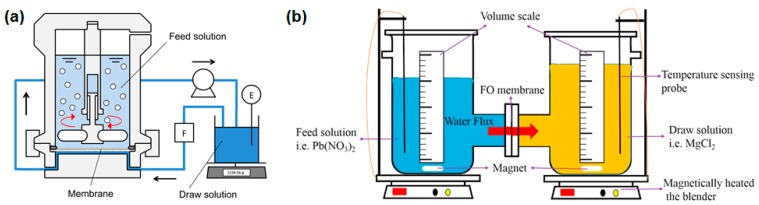
FO laboratory set-up (**a**) without FS circulation [48] and (**b**) without FS and DS circulation [49] (reprinted with permission from Elsevier).

Two different FO lab-scale set-ups are illustrated in Figure 6. Here, either the FS or both FS and DS are not circulated but stored directly above or beside the membrane. In the first published research paper dealing with FO application in manufacturing industry, a lab-scale set-up is described which is similar to the one illustrated in Figure 5. Only the DS and FS were not circulated but passed through the membrane test cell only once [42,43].

**Figure 7:**
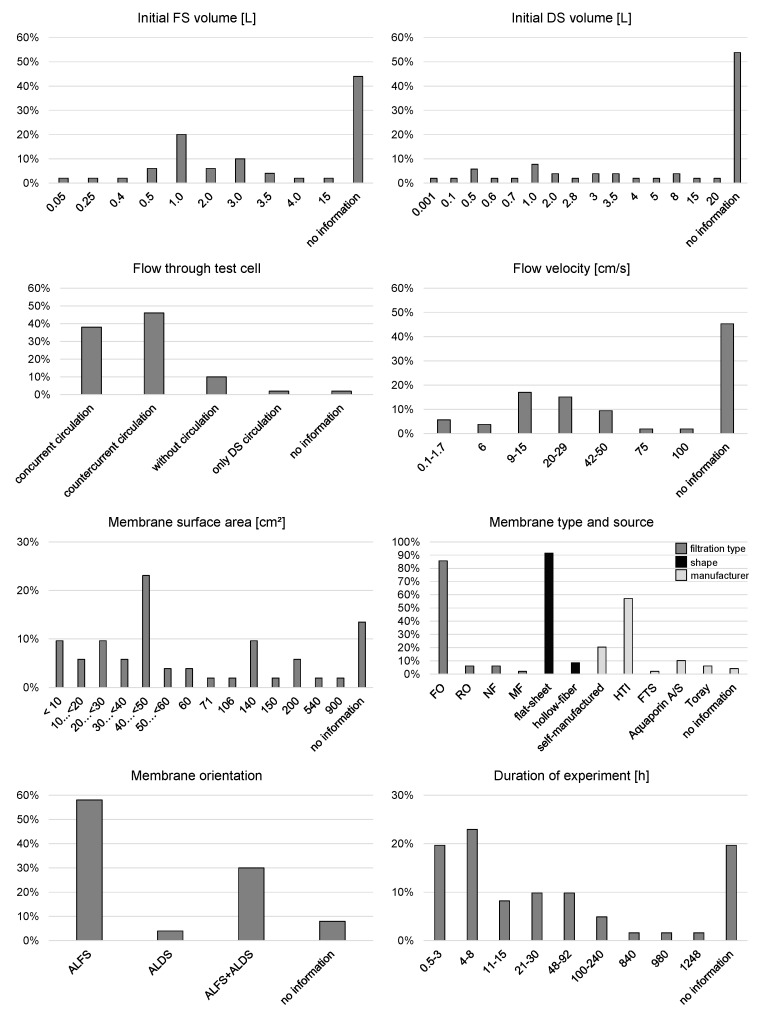
Evaluation of different FO lab-scale set-ups based on 51 original research papers.

Within this review paper, 51 original research papers were found and evaluated that dealt with FO application in manufacturing industry. All of these research results are based on lab-scale experiments. Figure 7 shows a summary of the different lab-scale set-ups comparing initial FS and DS volume, flow features through the membrane test cell, membrane characteristics, and duration of experiments.

Initial FS and DS volume was 0.05–15 and 0.001–20 L, respectively. The smallest initial DS volume of 0.001 L results from an experiment where coal powder was directly applied on the membrane surface [50]. The smallest initial liquid DS volume is 0.1 L. However, in 44% and 54% of the papers no information was given about the initial FS and DS volume, respectively.

As mentioned above, FO lab-scale set-ups usually have FS and DS circulation. Only 10% of the experiments were conducted without circulation. FS and DS were circulated concurrently in 38% and countercurrently in 46% of the included experiments. Flow velocity across the membrane was set to be between 0.1 and 100 cm/s. Mostly, flow velocities between 9 and 50 cm/s were chosen though. 45% of the papers did not give information about the flow velocity although it is an important parameter. Only the flow rate of FS and DS was given in many papers. However, without information about the flow channel dimensions, this parameter cannot be compared to other experiments.

The active membrane surface area ranged from 1.33 up to 900 cm^2^. Still, in 13% of the papers this information is missing. The majority of experiments were conducted with a membrane surface area between 40 and 50 cm^2^. Nearly all FO experiments used flat-sheet FO membranes. 20% of the membranes were self-manufactured and 76% were commercial membranes. HTI (Hydration Technology Innovations, LLC, Albany, OR, USA), a company, which to the authors’ knowledge has gone out of business, delivered most of the commercial membranes (57%). Other commercial suppliers were Aquaporin A/S (Kongens Lyngby, Denmark), Toray Korea Chemicals Inc. (Seoul, Korea), and FTS (Fluid Technology Solutions, Inc., Albany, OR, USA). The membranes were applied in ALFS mode in 58% or both in ALFS and ALDS mode in 30% of the experiments. Just 4% of the experiments were conducted with ALDS membrane orientation only.

Duration of experiments was reported to be between 0.5 and 1248 h. The majority of experiments lasted 0.5 to 8 h. The longest FO experiments lasting one month or longer were all related to biological processes like microalgae cultivation or biological wastewater treatment. The experiment duration is not exactly mentioned in 20% of the papers.

Due to the different lab-scale set-ups, the results of the papers cannot be compared easily. Especially, permeate fluxes are presented in different ways ranging from average permeate fluxes to start and end permeate fluxes. For this reason, permeate fluxes are not included in this summarizing evaluation but are given in the more detailed description in chapter 3 as well as in the Supplementary Material. The partial incomplete description of experimental parameters complicates repetition of experiments with comparable parameters, too. The parameters that should always be indicated are listed in Table 1.

### 2.2. Pilot-Scale

**Figure 8:**
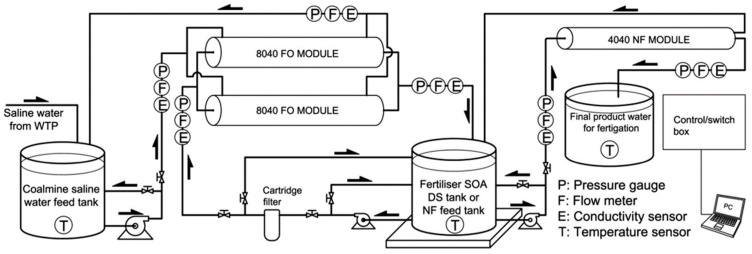
FO pilot-scale plant (reprinted from [51] with permission from Elsevier).

Pilot-scale investigations are necessary to prove the technical practicability of forward osmosis. One pilot-scale plant, which is shown in Figure 8, is operated by the working group of Prof. Shon in Sydney [51,52]. The overall layout is similar to the one for the lab-scale experiments. It contains two spiral wound FO membrane modules with one 8″ FO element each. The FO modules are operated in parallel. Different FO membrane modules have been used:

- two CTA FO modules from HTI (20.2 m^2^) [51],
- CTA FO module from HTI (9 m^2^) [52,53],
- TFC FO module from Toray Chemical Korea Inc. (15 m^2^) [52].

A nanofiltration module is intended for DS regeneration [51]. Although the system is designed for continuous operation, FO and NF have so far been operated batch-wise. During FO treatment, the volume of DS increases due to permeate flux. The volume of FS decreases but is kept constant by adding fresh FS continuously. Thus, the FS concentration increases slightly during FO operation. The volumes of the FS and DS tank are 5000 L.

The pilot-scale plant was used for experiments on FO application in fertigation, brackish water desalination and coal mining wastewater treatment [51,53]. It has not been used in manufacturing industries yet.

**Figure 9:**
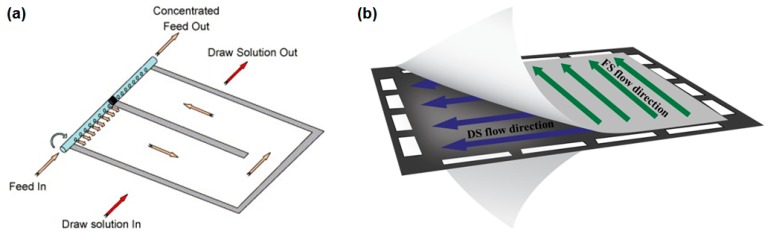
Flow channel configuration in (**a**) spiral wound FO element [4] and (**b**) plate-and-frame FO element (reprinted from [54] with permission from Elsevier).

In 2018 two studies have been published about pilot-scale investigations on plate-and-frame FO elements from Porifera, Inc. (Hayward, CA, USA) [54,55]. Proprietary flat-sheet membranes are arranged in membrane plates to enable a cross-current flow of FS and DS. Up to six elements can be combined in one module. A spacer in the FS channel is optional. The active layer of the membrane is facing the FS. These plate-and-frame elements have a lower packing density compared to spiral wound FO elements. Due to their simple flow channel configuration, wastewater with foulants or high viscosity is supposedly treated more easily. Figure 9 illustrates the flow channel configuration in a spiral wound FO element and a plate-and-frame FO element.

Song et al. used one single plate-and-frame FO element with a membrane area of 7 m^2^ consisting of 14 membrane sheets and performed regular FO experiments as well as pressure assisted FO experiments [54]. The experiments focused mainly on operating parameters, not on a special FO application case. Thus, mainly tap water was used as feed and draw solution, sometimes with an increased osmotic pressure. However, it is not mentioned which substance was used to increase the osmotic pressure of the tap water.

Lee et al. used three FO modules with one, three, and six plate-and-frame elements, respectively [55]. The membrane area of one element was 7 m^2^. Since the three and six element modules could be run in series, a maximum membrane area of 63 m^2^ was achieved. Tap water and sodium chloride solution with varying concentrations were the FS and DS to investigate different operating parameters. Real FO application was not investigated.

Within the research project “INSPIREWATER” funded by the European Union, lab-scale as well as pilot-scale studies on FO application in the chemical industry are conducted [56,57,58]. Chemical wastewater is first treated in an activated sludge plant. The effluent is then filtered by ultrafiltration and filtrate is lead to a reverse osmosis step. RO concentrate is further concentrated by FO using a synthetic DS (1 M NaCl, MgCl_2_, Na_2_SO_4_) which is regenerated by another RO step or membrane distillation. Furthermore, a second FO will be investigated for further concentration of concentrated FS from the first FO. So far, only results from lab-scale experiments have been published and are discussed in chapter 4.3 [57].

Information on pilot-scale FO application can also be found on websites from FO companies. Forward osmosis application in the semiconductor industry will be investigated on pilot-scale in a project run by the companies Darco Water Technologies Ltd. (Singapore) and Aquaporin (A/S) [59]. Based on a successful proof-of-principle study, semiconductor wastewater streams will be treated by FO in a pilot project.

An application of forward osmosis in dairy industry is the dewatering of raw milk. If raw milk was dewatered right after the milking process before transportation to the dairy plant, transportation costs and emissions could be reduced. The U.S. company Porifera Inc. (USA) report that they operated a pilot plant to treat 45,000 kg/h milk with their patented FO system [60]. The milk was concentrated 4 times producing 11,250 kg/h milk concentrate. Compared to a thermal evaporation process, FO could save 44% energy, 24% steam, 80% investments costs (CAPEX), and 50% operating costs (OPEX). Forward osmosis application in a dairy from ARLA Food in Denmark is currently investigated on pilot-scale, as reported by the Danish company Aquaporin A/S [61]. Unfortunately, no detailed information is given.

### 2.3. Industrial Scale

So far, FO application for industrial scale has rarely been reported. The British company Modern Water plc reports on its website about building a commercial FO plant in Al Khaluf (Oman) where seawater is desalinated for drinking water purposes. Another Modern Water seawater desalination plant (500 m^3^/d) is being constructed and will start operation in the beginning of 2018 [62,63,64]. Oasys Water Inc. (Cambridge, MA, USA) states on its website the construction of a commercial FO plant to treat 630 m^3^/d wastewater from a power plant [65]. In combination with other treatment technologies, a zero liquid discharge (ZLD) concept shall be realized.

There are a few companies worldwide offering commercial FO systems. They are (in alphabetical order):

- Aquaporin A/S (Kongens Lyngby, Denmark) [66,67],
- Aquaporin Asia Pte. Ltd. (Singapore) [68,69],
- BLUE-tec BV (Renkum, The Netherlands) [56,70],
- Darco Water Technologies Ltd. (Singapore) [71,72],
- De.mem Ltd. (Singapore) [73],
- Fluid Technology Solutions, Inc. (FTS, Albany, OR, USA) [74],
- Hydration Technology Innovations, LLC (HTI, Albany, OR, USA)—meanwhile out of business [52],
- Modern Water plc. (London, UK) [62,63,64],
- Oasys Water, Inc. (Cambridge, MA, USA) [75,76],
- Porifera, Inc. (Hayward, CA, USA) [55],
- Toray Chemical Korea, Inc. (Seoul, Korea) [52],
- Trevi Systems, Inc. (Petaluma, CA, USA) [77],
- W.O.G. Technologies Pte Ltd. (Singapore) [69].

### 2.4. Fields of Forward Osmosis Application

Investigation of forward osmosis application ranges from lab-scale experiments (with either synthetic or real water) to full-scale implementation (with real water) and covers many fields, including:

- seawater desalination to produce drinking water [62,63,64],
- emergency water supply with so-called hydration bags [78],
- treatment of wastewater from oil and gas production as well as from mining [34,79,80,81,82],
- agricultural use for fertigation [83,84,85],
- biological wastewater treatment with osmotic membrane bioreactors [37,86,87,88,89],
- treatment of anaerobic digester centrate [90,91],
- microbial fuel cells [92,93,94,95,96,97,98],
- removal of trace organic compounds [99,100,101,102,103,104].

As can be seen, different types of water are subject for FO application. Another field of FO application might be the treatment of industrial effluents and wastewaters as they occur in manufacturing industries. Here as well as in other applications, energy efficient and economic treatment technologies are of great interest.

## 3. Application of Forward Osmosis Technology in Manufacturing Industries

### 3.1. Overview

The first research about FO application related to industries was published in the 1970s [42,43]. Here two lab-scale plants (13 and 58 cm^2^ membrane surface area) and a pilot-scale plant were constructed. Deionized water, copper solution, chromium solution, and wastewater from a fish and shell fish processing plant were used as FS. DS were synthetic seawater and concentrated sugar solution. 10 commercial RO membranes and one self-manufactured CTA membrane were tested. FO experiments were run without FS and DS circulation. Since the highest permeate flux in the lab-scale experiments was only 4.5 L/(m^2^·h), no pilot-scale experiments were conducted. Substance diffusion through the membrane, either from DS into FS or from FS into DS, was very high. The research project was stopped because no suitable FO membranes were available.

From 1977 until 2004, no articles were published about forward osmosis. Since 2004, FO research and publications have increased rapidely. FO technology was further developed and first industrial scale applications were realized, e.g., in seawater desalination.

Because of its benefits, FO technology might also be beneficial for industrial wastewater treatment. This paper gives an overview about the current FO application in industries, focusing on manufacturing industries. The following branches are included:

- food and beverage industry,
- chemical industry,
- pharmaceutical industry,
- coal processing industry,
- micro-algae cultivation,
- textile industry,
- pulp and paper industry,
- electronic industry,
- car manufacturing industry,
- industries with heavy metal usage.

**Figure 10:**
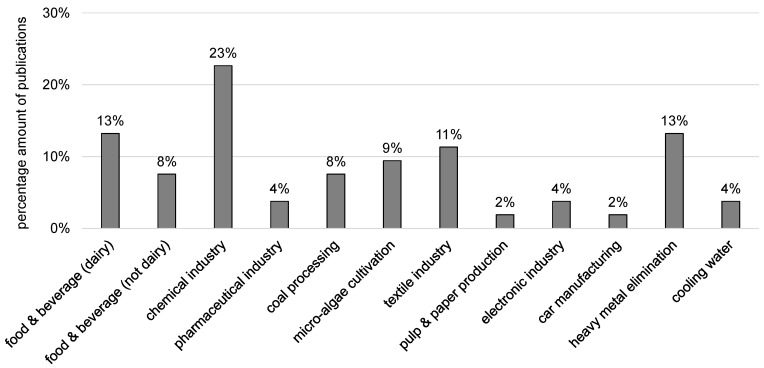
Industrial branches of evaluated research papers and percentage distribution.

All in all, 51 original research papers were identified and evaluated. FO desalination for the production of drinking water was not included because there is only little relation to manufacturing industry. Other review papers have already described the concentration of fruit and vegetable juices by FO. Therefore, this topic is only briefly mentioned here. Figure 10 illustrates the percentage distribution of the 51 papers on the different branches.

Most articles were published about FO application in chemical industry. Heavy metal elimination by FO was also often addressed and included in this review because heavy metals are often present in industrial wastewaters. Many articles were found about FO application in the food and beverage industry including dairy industry. Here, FO was mainly used to treat the products (milk, juice, whey) but also wastewaters.

More information on the individual applications are given in the following chapters of this review focusing on implemented feed and draw solutions, resulting permeate fluxes, and applied hybrid technologies. A summary with more details can be found in the Supplementary Material of this article.

### 3.2. Food & Beverage Industry

#### 3.2.1. Dairy Industry

Dairy industry uses raw milk to produce several food items like long-life milk, cheese, and yogurt. Large amounts of wastewater result from the manufacturing from either cleaning procedures or dewatering processes [105]. Usually, this wastewater is treated before disposal. Wastewater recycling or reuse is also an issue in dairy industry.

Several research results were published about FO treatment for whey dewatering. During cheese manufacturing, whey is a waste product that is nowadays further processed into valuable products e.g., whey powder. Pressure-driven membrane processes and evaporation processes are used to dehydrate raw whey. However, those conventional processes consume a lot of energy. Forward osmosis might be applicable for energy-efficient whey dewatering.

The concentration of dairy whey with forward osmosis was investigated in several studies in Turkey [106,107,108,109,110,111]. Raw whey from a cheese manufacturing was used as FS. The DS were either NaCl (2 M or 3 M) or NH_4_HCO_3_ (2 M). In lab-scale experiments, the performance of different whey processing technologies was measured and compared. Economic evaluation was also included in the studies. The technologies for whey dewatering and—in case of applied FO—corresponding DS regeneration were:

- ultrafiltration and reverse osmosis (UF-RO) [106,108],
- forward osmosis with reverse osmosis (FO-RO, NaCl as DS) [106,108,109],
- forward osmosis with reverse osmosis (FO-RO, 2 M NH_4_HCO_3_ as DS with thermal enhanced DS regeneration) [106],
- forward osmosis with membrane distillation (FO-MD, 2 M NaCl as DS) [106,107],
- membrane distillation and reverse osmosis (MD-RO) [106,107].

**Figure 11:**
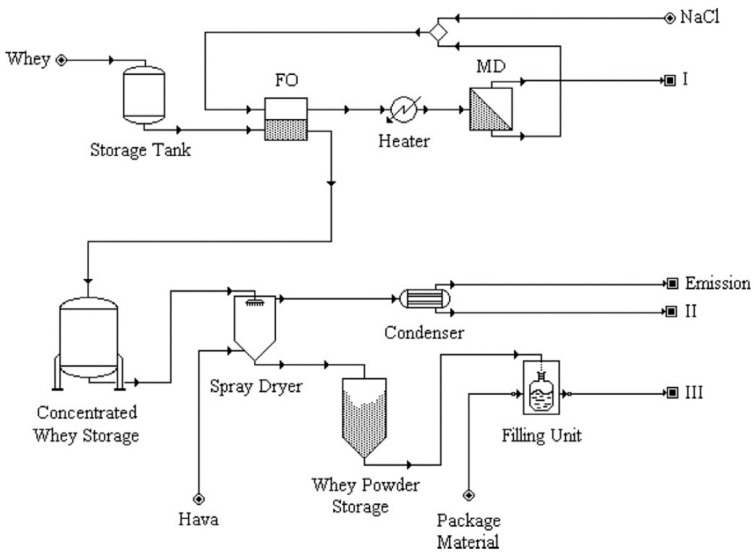
FO-MD application scenario for dairy whey dewatering (I water recovery line, II centralized wastewater treatment line, III packaged whey powder product line) (reprinted from [107] with permission from Elsevier).

They found that, if waste heat was available, FO-MD and MD-RO were the most economic treatment technologies for whey dewatering. Without waste heat usage, FO-RO and MD-RO were the recommended treatment option. Figure 11 illustrates the proposed FO-MD application for whey dewatering.

Wang et al. reported about FO lab-scale experiments with artificial whey solution obtained by mixing whey powder with deionized water [112]. The 6% whey solution was the FS; NaCl the DS (mostly 0.5 M, but also 0.3 and 1.0 M). A self-manufactured hollow-fiber membrane was used. Permeate fluxes were between 9.5 and 14 L/(m^2^·h) in the beginning of the experiments and decreased by 11% during the experiments.

Pal et al. reported about sweet cheese whey which was separated into whey lactose for acetic acid manufacturing by fermentation and whey protein solution for the production of whey protein powder [113]. FO was investigated to be implemented in two ways. First, FO was used as pretreatment step before fermentation. Sweet cheese whey was either treated by MF and the permeate was then used as FS in FO treatment, or sweet cheese whey was filtered by MF und concentrated by UF and then used as FS in FO treatment. Thereby, whey protein solution could be concentrated before drying. Second, FO was used as fermentation follow-up treatment for concentrating the acetic acid that was separated from the fermentation broth by NF. In both cases, the DS was 1 M MgSO_4_ and NF was the DS regeneration technology. Without pressure, FO permeate fluxes were 19 and 25 L/(m^2^·h) for whey dewatering and acetic acid dewatering, respectively. If external pressure was applied (up to 2 bar), this pressure-enhanced osmosis enabled higher permeate fluxes up to 42 and 44 L/(m^2^·h). Protein and acetic acid rejection was between 71% and 84%. Pal et al. (2016) also evaluated the economic factors comparing the suggested FO including technology with a conventional system. They found that FO requires less energy and less space resulting in lower costs.

The hybrid system of FO and MD was investigated from Song et al. [114]. They used real dairy wastewater as FS and NaCl as DS as well as two different FO membranes. As a result, FO could concentrate real dairy wastewater and MD could obtain desalted water. FO permeate fluxes were between 10.7 and 3.5 L/(m^2^·h) in the FO experiments without DS regeneration and between 18 and 6 L/(m^2^·h) in FO-MD hybrid experiments. Fouling of the FO membrane occurred, but membrane cleaning (rinsing with deionized water and osmotic backwash) could restore the membrane performance up to 90%.

Another study investigated if RO concentrate from a dairy wastewater treatment plant was suitable as FS and if cheese brine could be used as DS in forward osmosis [115]. Dairy cheese brine proved to be a good DS: average permeate flux was 21.0 L/(m^2^·h) with deionized water as FS. RO concentrate was further concentrated by FO with an average permeate flux of 7.9 L/(m^2^·h) when 1 M NaCl was used as DS. When FS was RO concentrate and DS was cheese whey, the average permeate flux was 15.1 L/(m^2^·h). This shows that—with FO—a combined treatment of both wastewaters is possible and thus no separate DS regeneration is necessary.

#### 3.2.2. Juice Processing

Fruit and vegetable juices are often concentrated to reduce its volume and safe transportation and storage costs. Moreover, natural colorants are obtained from fruit juices by concentration. This concentration is conventionally done by pressure-driven membrane filtration processes like reverse osmosis or thermally driven evaporation [35]. In both cases, the composition and characteristics of the juice (color, flavor, and nutritional compounds) might be negatively influenced. Forward osmosis possibly concentrates juice without high pressures and without heating. Thus, the characteristics of the juice remains unchanged.

There are numerous publications on juice concentration by FO. Rastogi et al. [35] summarize the state of knowledge up to January 2016. They report about the FO concentration of grape juice, tomato juice, pineapple juice, and raspberry juice. Furthermore, they describe that FO concentrates plant-based colorants (anthocyanin extract from red radish or kokum, betalain extract from beetroot), orange peel press liquor, and artificial sugar solutions. All results are based on lab-scale FO experiments though. Juices were concentrated up to a sugar content of 30 to 60° Brix. Reported FO permeate fluxes were between 2.5 and 9.1 L/(m^2^·h). Draw solutions were NaCl, CaCl_2_, Ca(NO_3_)_2_, sucrose solution, fructose solution, high fructose corn syrup, and polyethylene glycol.

#### 3.2.3. Other Food & Beverage Application

Marques et al. investigated FO to produce tea extracts [116]. Although the process is called osmotic evaporation and a hollow-fiber membrane contactor is used for experiments, the operating principle is the same as forward osmosis. Tea is used as FS and 5 M CaCl_2_ as DS. Within 5 h, a tea concentration of 40% was obtained. Permeate flux could be kept constant apart from the decrease due to declining osmotic pressure difference.

**Figure 12:**
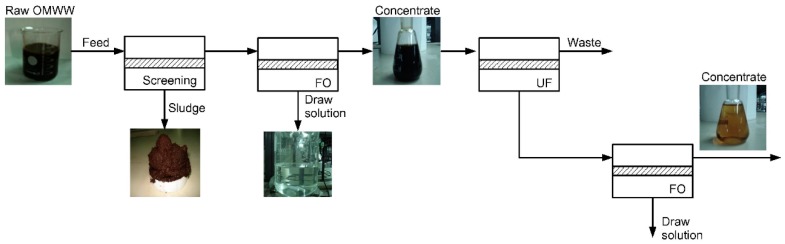
FO application for the treatment of olive mill wastewater (reprinted from [117] with permission from Elsevier).

FO treatment of olive mill wastewater was studied by Gebreyohannes et al. [117]. They used real wastewater which is rich in biophenolic compounds as FS and MgCl_2_ as DS. Long-term experiments were conducted for 8 days, in which FS and DS were refreshed daily. FO permeate flux was between 9.8 and 7.1 L/(m^2^·h). Fouling was observed but pure water permeability could be restored to 95% by rinsing and osmotic backwashing. All in all, volume reduction was 71%. Different pre-treatment methods were tested for the wastewater. Particle retention by microfiltration increased FO permeate flux. Biological treatment in a membrane bioreactor combined with microfiltration even further enhanced FO permeate flux because pectins in the wastewater were reduced by 92%. The concentrated wastewater after FO was treated by ultrafiltration. UF permeate was rich in low molecular biophenols and used as FS in a second FO with MgCl_2_ as DS. Here, FO permeate flux was 5 L/(m^2^·h) and volume reduction was 64%. Figure 12 illustrates the proposed treatment chain.

Singh et al. examined the FO concentration of distillery wastewater [118]. They used real wastewater from sugarcane molasses distillery as FS and MgCl_2_ as DS. FO permeate flux was only 2.8 L/(m^2^·h), which is low compared to the permeate fluxes with olive mill wastewater mentioned above. Still, water recovery after 24 h was 70%, which would be higher than with RO (35–40%). Rejection and permeate flux was stable over five 24 h experiments with the same membrane that was rinsed with water in between.

**Figure 13:**
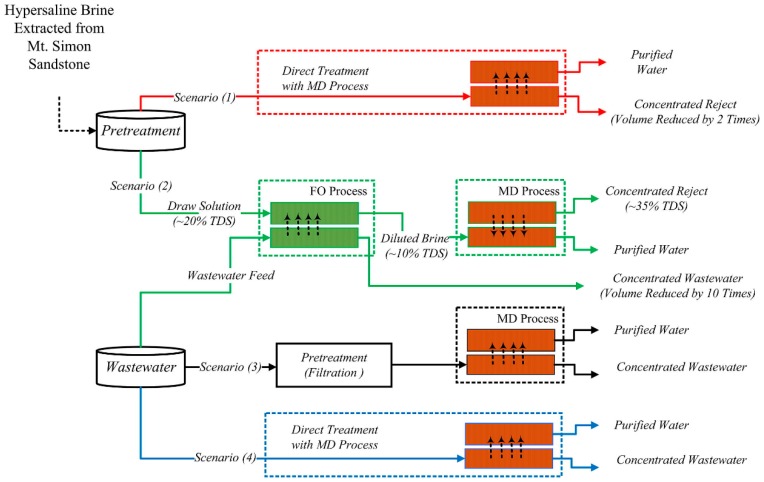
FO application and other treatment possibilities for grain processing wastewater and brine from CO_2_ sequestration site (reprinted from [119] with permission from Elsevier).

Salih et al. used wastewater from a grain processing plant as FS in FO process [119]. This wastewater was first treated biologically and by dissolved air flotation. The DS was hypersaline brine from a potential CO_2_ sequestration site. FO permeate fluxes were between 10 and 15 kg/(m^2^·h). The brine produced higher FO permeate flux than 20% MgSO_4_ as DS but also higher reverse salt flux. Different treatment options for both wastewater and brine were evaluated (Figure 13). FO or MD (either with or without pre-filtration) concentrated grain processing wastewater. Purified water from the brine was gained by MD or FO-MD (brine being the DS regenerated by MD). Treatment options with FO had the advantage that fouling was reversible.

FO can also be used to produce drinking water from seawater. In this application, seawater is the FS and a highly concentrated solution is the DS. A regeneration technology concentrates the diluted DS and produces drinking water. An overview about FO seawater desalination is provided in several reviews elsewhere [28,29,30,35].

### 3.3. Chemical Industry

Wünsch et al. investigated the FO treatment of secondary effluent from an industrial wastewater treatment plant [57]. Based on the list of co-authors it is likely that the wastewater originates from chemical industry. The secondary effluent was first concentrated by UF (85%) and RO (50%). Afterwards a softening step was applied (soda ash treatment). The resulting wastewater was then used as FS in in lab-scale FO experiment. Here, three different DS were evaluated (NaCl, Na_2_SO_4_, MgCl_2_) all having the same concentration (1 mol/L). Thus in the FO experiment, the osmotic pressure difference was not equal but was 115 bar, 33.4 bar, and 35.1 bar with MgCl_2_, Na_2_SO_4_, and NaCl, respectively. Permeate fluxes for 67% water recovery were interpolated from measured data. They were 13.0 L/(m^2^·h), 8.08 L/(m^2^·h), and 9.63 L/(m^2^·h) with MgCl_2_, Na_2_SO_4_, and NaCl, respectively. MgCl_2_ was the best DS because it delivered the highest permeate flux and lowest reverse salt flux.

Wastewater from esterification was treated in another study [120]. It was pretreated and used as FS in FO experiments with different self-manufactured CTA FO membranes. Within the first 5 h, permeate flux declined from 9.56 L/(m^2^·h) to 6.0 L/(m^2^·h). Afterwards, it declined slower which is probably due to a stable fouling layer on the membrane surface. TOC rejection was very high (>96%) and water recovery was 57.1%.

**Figure 14:**
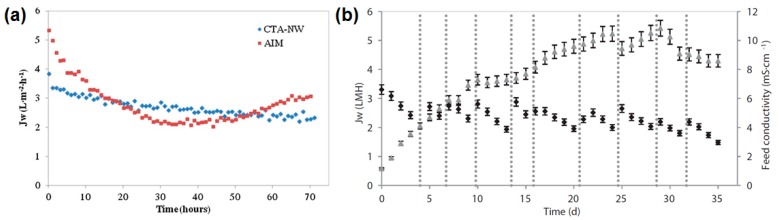
FO permeate flux with wastewater from ammonia absorption as DS and (**a**) anaerobically digested sludge centrate as FS [121] or (**b**) activated sludge as FS (OMBR) [87] (reprinted with permission from Elsevier).

Two studies used wastewater from industrial ammonia absorption processes as DS in FO process [87,121]. This wastewater has high sulfate and ammonia concentrations and therefore a high osmotic pressure. The acidic pH was adjusted to pH 7 or pH 4 so that the membrane in the lab-scale experiments was not damaged. In one of the studies, anaerobically digested sludge centrate from a municipal wastewater treatment plant was used as FS [121]. Permeate fluxes here were between 2 and 5 L/(m^2^·h) (Figure 14a). Nitrogen in the sludge centrate could be concentrated successfully. Phosphorus concentration, however, was not successful because it precipitated as calcium phosphate. In the other study, an osmotic bioreactor was simulated and activated sludge was the FS in the FO process [87]. Permeate fluxes were between 1 und 3 L/(m^2^·h) (Figure 14b). Osmotic backwash was applied regularly to clean the membrane. In both cases, wastewater from ammonia absorption was a good DS.

An interesting FO application is proposed by Takahashi et al. [48]. They use FO to dehydrate polyvinyl chloride (PVC) latex before it is dried. Unlike in most other lab-scale FO experiments, the FS was not circulated through the membrane test cell but was placed in a reservoir above the membrane (Figure 6). Synthetic seawater (0.8–1.8 M NaCl) was the DS. Permeate fluxes in the beginning of the experiments were 8 and 4.5 L/(m^2^·h) depending on the membrane type. After 24 h the PVC latex concentration reached 75 wt %. However, cake layer formation occurred in the end. For this reason, a final PVC concentration of 60 to 64 wt % is proposed.

**Figure 15:**
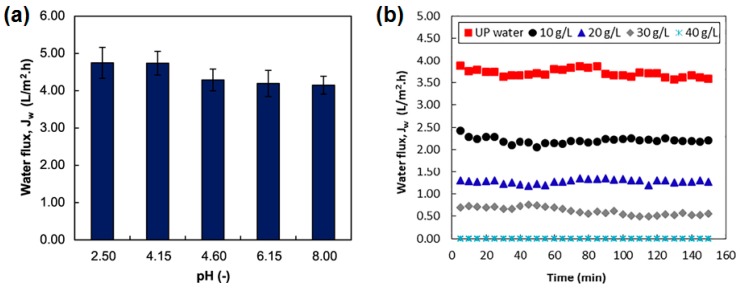
FO permeate fluxes with (**a**) 20 g/L succinic acid as FS and 1 M NaCl as FS; (**b**) succinic acid as FS (pH 6.9) and real seawater as DS (reprinted from [122] with permission from Elsevier).

The application of forward osmosis combined with biological fermentation processes was subject in many studies. Law et al. used succinic acid as FS combined with seawater as FS [122]. Succinic acid is raw material for many chemical production processes and is traditionally produced from petroleum. Another way to obtain succinic acid is fermentation. Here, the succinic acid has to be eliminated from the fermentation broth and further concentrated. FO was examined to concentrate succinic acid depending on its pH. Furthermore, real seawater was used as DS. Figure 15 shows the permeate fluxes which were between 0 and 4.8 L/(m^2^·h). A patent was issued on the FO concentration of fermentation broths [123]. Here, succinic acid (67 g/L) was the FS and NaCl (30 wt %) was the DS. FO application in fermentation broth treatment was also investigated in another study [124]. In this case, butyric acid was used as FS and MgCl_2_ as DS. Permeate flux varied between 16 and 18 L/(m^2^·h).

**Figure 16:**
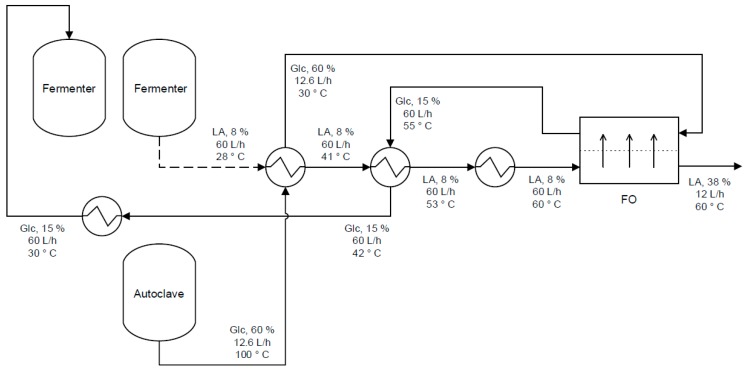
Proposed FO application with glucose (Glc.) as DS in production of lactic acid (LA) by fermentation (reprinted from [125] with permission from author).

Ihalainen describes in her master thesis the FO treatment of lactic acid [125]. Lactic acid, like succinic acid, can be produced by fermentation requiring post-treatment e.g., concentration. FO experiments were conducted with lactic acid and glucose as FS and DS, respectively. In the long-term experiment, the permeate flux was 12 L/(m^2^·h) corresponding to 84% water recovery. However, lactic acid rejection was only 56% meaning that valuable product is lost. The diluted glucose solution can be used as carbohydrate source for the fermentation process (Figure 16). Thus, no DS regeneration is necessary for this application.

**Figure 17:**
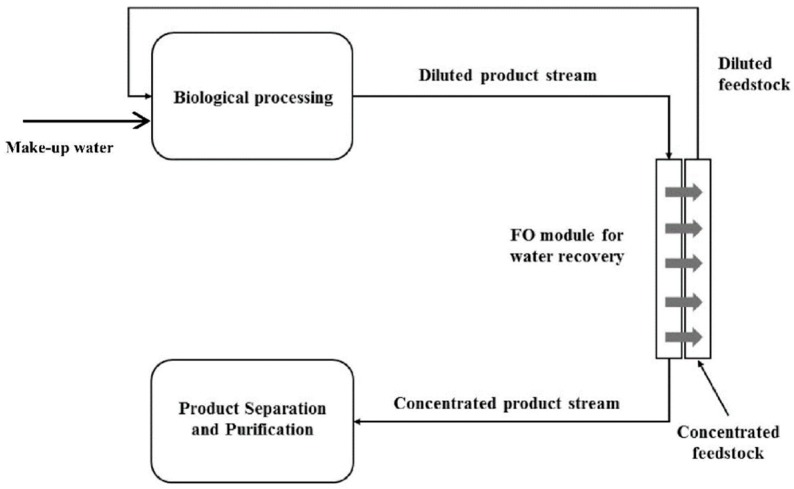
Proposed FO application with fermentation feedstock concentrate as DS and fermentation product as FS (reprinted from [126] with permission from Elsevier).

A similar FO application concept was proposed by Kalafatakis et al. [126]. Crude glycerol as well as pretreated and enzymatically hydrolysed wheat straw (PHWS) were the investigated DS. After dilution in FO process, they are transferred in the fermentation reactor as feedstock. DS regeneration is not necessary. The corresponding FS is the fermentation broth, which is concentrated in the FO process (Figure 17). In the experiments, however, FS was created by using the same substance as the DS highly diluted with deionized water. With crude glycerol (100%) as DS, permeate fluxes were 8.4, 9.0 and 10.5 L/(m^2^·h) with FS glycerol concentrations of 5, 2, and 1%, respectively. When 100% PHWS was the DS, permeate fluxes were 1.3, 5.4, and 6.2 L/(m^2^·h) with FS PHWS concentration being 20, 5, and 0%, respectively. Permeate fluxes were calculated from the first 30 min of the experiments. In addition to the lab-scale experiments, the usage of crude glycerol as DS and its fermentation to produce butanol was economically evaluated. As a result, they showed that 50% water reclamation could reduce butanol purification costs by 50%.

So far, FO was applied to concentrate the fermentation product stream. Shibuya et al. investigated FO to concentrate the fermentation feedstock [127,128]. In both studies, ethanol was produced by fermentation from lignocellulose biomass. The sugar-containing liquid fraction from rice straw pretreated with hot water was used as FS or simulated by using a synthetic sugar solution. Before FO, the liquid was filtered. So-called switchable polarity solvents (SPS) were used as DS. SPS can be mixed with water when CO_2_ is present. In the absence of CO_2_, they separate from water. Thus, DS regeneration can be accomplished easily.

**Figure 18:**
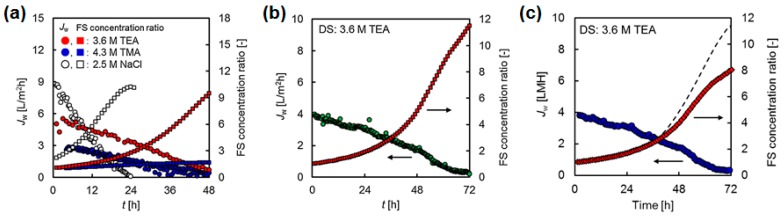
FO permeate flux with FS being model sugar solution (**a**), liquid fraction of pretreated rice straw after filtration (**b**) and filtration and enzymatic hydrolysis (**c**) (reprinted from [127,128] with permission from Elsevier).

In the first study, FO successfully concentrated the sugar solution as well as the liquid fraction of the pretreated rice straw. Nevertheless, fermentation inhibitors were also concentrated [127]. For this reason, different treatment technologies were combined and investigated in the second study [128]. Here, the sugar containing solution was to be concentrated whereas the inhibitors were supposed to be removed. NF concentration with water addition was performed before enzymatic hydrolysis and FO treatment. Experiments showed that this treatment chain delivered a high ethanol yield in the fermentation process. Permeate fluxes varied between 0.8 and 9 L/(m^2^·h) (Figure 18).

Several researchers focused on the FO treatment of acids. In one study, different carboxylic acids were concentrated by FO [129]. These acids are utilized in many chemical processes. For this reason, they are likely to be contained in the wastewater. Acetic, butyric, valeric, and lactic acid (concentration 10 mM) were the FS in the FO experiments. Ammonium chloride was the DS. A model was developed to simulate the FO experiments. The comparison of the results showed that they matched well proving the correctness of the proposed model. Taken the average weight change of approximately 0.6 kg within the 30-h experiment and a membrane surface area of 42 cm^2^, the permeate flux was 4.8 L/(m^2^·h) for all tested acids.

### 3.4. Pharmaceutical Industry

Closely related to the chemical industry is the pharmaceutical industry. Two research papers published results from forward osmosis experiments treating pharmaceutical liquids. Cui et al. (2018) reported about FO experiments in which they used typical pharmaceutical solvents as FS, which usually contain pharmaceutical active ingredients (API) [130]. The aim was to recover the organic solvents and reject the API. So, in this case, not water but organic solvents were supposed to pass through the membrane and dilute the DS. Ethanol, isopropanol, and hexane were the tested FS, in some cases with dissolved tetracycline and triglycerides. DS were lithium chloride, methyl palmitate, citric acid, polyethylene glycol, and diethanolamine. Average solvent fluxes were between 0.32 ± 0.07 and 3.82 ± 0.37 L/(m^2^·h). API rejection was >98%.

Wang et al. investigated the concentration of protein solutions by forward osmosis [131]. They used bovine serum albumin (BSA) solution as FS and NaCl as DS. Membrane distillation was applied for DS regeneration and self-manufactured hollow-fibre membranes were used for FO and MD. Initial permeate fluxes were 2.7 and 5.3 L/(m^2^·h) with 0.5 and 2 M NaCl, respectively.

### 3.5. Coal Processing

FO is potentially applicable for the treatment of wastewater from mining [80]. A further step would be to investigate FO application in coal processing industry. Kumar et al. investigated a hybrid system of FO and NF to recycle coke-oven wastewater [132]. The wastewater came from a factory that produces coke for steel manufacturing. Coke-oven wastewater usually contains toxic substances [133]. For this reason, it has to be treated before disposal. According to Kumar et al. biological treatment, adsorption, coagulation, wet oxidation, and advanced oxidation processes have been examined as treatment technologies so far [132]. However, all of these technologies are either technically or economically difficult. In lab-scale forward osmosis experiments, Kumar et al. used real coke-oven wastewater as FS. NaCl, MgSO_4_, and CaCl_2_·H_2_O (0.4–2.5 M) were the DS. FO permeate fluxes were 42–46 L/(m^2^·h) and substance rejection exceeded 97%. NF was operated simultaneously to concentrate and recycle the DS. Overall, the hybrid system worked well. Occuring fouling proved to be reversible. Economic calculations showed that FO-NF would be an economic alternative to other treatment options. Fenton oxidation processes or struvite precipitation could further treat concentrated coke-oven wastewater after FO.

The treatment of coal gasification wastewater with FO was investigated in a different study [134]. This wastewater is hard to treat because it contains toxic phenolic compounds. In FO experiments, three types of artificial coal gasification wastewater (100 mg/L of three phenolic compounds, various pH-values) and sodium chloride (1.75–10.5%) were the FS and DS, respectively. It was found that coal gasification wastewater could be concentrated by FO. Rejection of phenolic compounds was better with alkaline pH-values and higher DS concentration, which also increased the permeate water flux. Permeate water fluxes varied between approximately 8.5 and 10.5 L/(m^2^·h). The authors of the study also developed a model to represent their experiments. Simulated results and experimental results matched well supporting the established model. The focus of this study was the rejection of phenolic compounds by FO membrane. Regarding further treatment technologies, e.g., the concentrated coal gasification wastewater or the diluted NaCl solution, no suggestions are made.

Another study investigated the rejection of phenol by different forward osmosis membranes [74]. The FS was an artificial wastewater from oil and gas industry. It contained phenol and sodium chloride. Sodium chloride (0.5–4 mol/L) was the DS. The sorption and the rejection of phenol varied depending on the operation conditions and on the three different membrane types used. Furthermore, a model was established and validated. In general, the results of this study correspond to the ones mentioned above [134].

### 3.6. Micro Algae Cultivation

Algal biomass has drawn raising attention because it provides multiple benefits. Microalgae are considered a renewable energy source, e.g., for biofuel production [135]. Furthermore, industries like food and cosmetic industries use microalgae as raw material for their products [136]. During the cultivation of microalgae, a large quantity of substances in the surrounding water is consumed and CO_2_ can be captured [137]. For this reason, microalgae cultivation not only provides a valuable product but can also be used for wastewater treatment. However, the separation of microalgae from water is an economically critical issue. Different treatment methods have been investigated including centrifugation, flotation, flocculation, sedimentation, and pressure-driven membrane processes [138,139]. Forward osmosis might be an alternative treatment technology here.

Larronde-Larretche et al. concentrated different microalgae solutions with FO [140]. Three different DS were used: sea salt solution, MgCl_2_, and CaCl_2_. DS concentration was set to provide the same initial permeate flux of 7 L/(m^2^·h). FO experiments were conducted until permeate volume was 75% of initial FS volume. Permeate flux declined in the course of the experiments. The extent of this flux loss varied between 5 and 71% depending on the microalgae species and the used DS. Algae dewatering efficiency was between 59 and 80%. For technical application, the usage of seawater as DS was proposed either before seawater desalination to facilitate desalination reverse osmosis or after seawater desalination to dilute RO concentrate before sea disposal. In this case, DS regeneration is not necessary.

**Figure 19:**
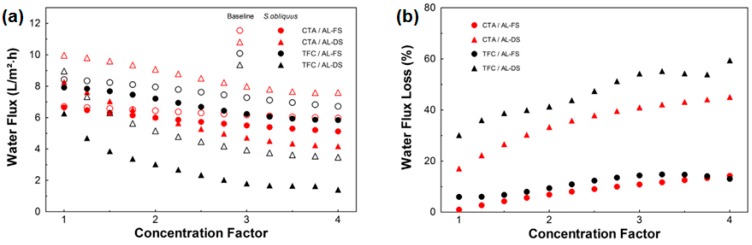
FO permeate flux (**a**) and permeate flux loss (**b**) with 0.2 g/L microalgae suspension as FS and 70 g/L sea salt solution as DS (reprinted from [141] with permission from Elsevier).

In a previous study, Larronde-Larretche et al. also investigated FO concentration of microalgae [141]. One microalgae species solution was the FS. The DS were sea salt solution, NaCl, MgCl_2_, and CaCl_2_. It was shown that ALFS membrane orientation was more suitable because permeate flux loss was lower than in ALDS membrane orientation and fouling layers were easily removable by rinsing with deionized water. NaCl was the best DS followed by MgCl_2_. If calcium was present in the DS more severe fouling occurred and permeate flux decreased a lot. Permeate fluxes in the beginning of the experiment were 6.7–8.2 L/(m^2^·h), after 75% permeate volume they were 1.5–5.9 L/(m^2^·h). Permeate flux losses were between 10 and 59% (Figure 19).

If FO is used for microalgae concentration, fouling is a critical point to be considered. In the studies mentioned above, fouling occurred resulting in permeate flux loss. However, it is mentioned that fouling could be minimized by choosing proper microalgae species and DS type. Furthermore, fouling was reversible. These facts are supported by other studies [142,143]. Here, it was shown that magnesium ions in the DS enhance fouling formation and make it harder to be removed. With NaCl as DS less fouling occurred and was also reversible. Spacers in the FS feed channel further reduced the negative impact of fouling on FO performance.

Buckwalter et al. proposed a different algae cultivation method [144]. Algae were not cultivated inside a bioreactor but inside a membrane bag filled with nutrient solution. The bags were stored in the sea. This way, microalgae growth and concentration by FO process took place at the same time. The bags were taken out of the sea and algae were harvested. The membrane bags were based on the so-called hydration bags and made of FO CTA membrane from HTI. The nutrient-algae solution inside the bags was the FS and seawater the DS in FO process. Average permeate flux was 2 L/(m^2^·h). Fouling occurred but did not affect algae dewatering. However, membrane bags were damaged in long-term experiments in the ocean.

### 3.7. Textile Industry

Manufacturing of textiles is an industry with a high water demand of 200 to 400 L per kg produced fabric [145]. Especially, dying and conditioning technologies use large amounts of water and produce wastewater that has to be treated [146]. Wastewater treatment and water recycling can enable a more sustainable production process. Physico-chemical processes (coagulation, flotation, chemical oxidation, and biodegradation) as well as advanced treatment technologies (adsorption, ozonation, photocatalysis, and membrane processes) have been investigated for textile wastewater treatment [147].

**Figure 20:**
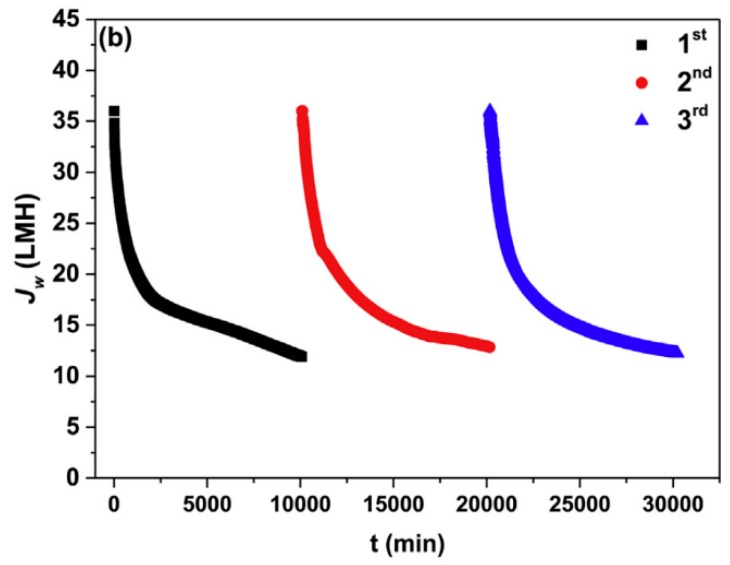
Results of FO experiment with synthetic textile wastewater as FS and 2 M NaCl as DS (TFC-FO-membrane, self-manufactured; ALFS; membrane flushed with DI water between test runs) (reprinted from [148] with permission from Elsevier).

Han et al. propose the application of forward osmosis to treat textile wastewater [148]. FO shall concentrate the dye-containing wastewater as FS. Subsequently, the concentrate is to be treated by coagulation and flocculation. In lab-scale experiments, different synthetic dye wastewaters were tested. DS was sodium chloride (2 mol/L). Dye retention was almost 100%. Permeate flux in the beginning was 36 L/(m^2^·h), decreased to 12 L/(m^2^·h), and was maintained at this level. Fouling occurred but was reversible by rinsing with water (Figure 20).

**Figure 21:**
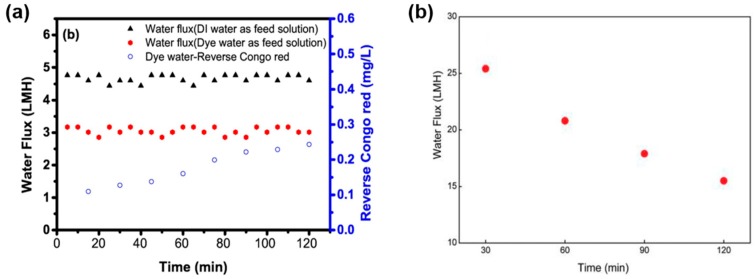
Results of FO experiment with (**a**) DI water or dye water (50 ppm Congo red aqueous solution) as FS and 0.25 g/mL P(SSA-co-MA)-Na-1 as DS (TFC-FO-membrane from HTI; ALFS) (reprinted from [149] with permission from Elsevier) and (**b**) dye water (50 ppm Acid Orange 8 aequeous solution) as FS and 0.48 g/mL mL PAA-Na as DS (self-manufactured hollow fiber FO membrane; ALDS) (reprinted from [150] with permission from American Chemical Society).

Other studies also investigated the FO application to concentrate dye-containing textile wastewater focusing on the type of draw solution. Polyelectrolytes and brown coal slurry were tested [50,149,150]. Huang et al. [149] used different polyelectrolytes as DS and dye containing wastewater as FS (50 ppm Congo red aqueous solution). They showed that polyelectrolyte P(SSA-co-MA)-Na-1 as DS has the advantage—besides its high osmotic pressure—to be regenerated easily by nanofiltration because of its large molecular size. Rejection rate of Congo red was high, although TOC in the DS increased a little bit. Average permeate flux in the 2 h FO experiment with the mentioned FS and DS was ca. 3 L/(m^2^·h) (Figure 21a). Ge et al. also used polyelectrolyte as DS [150]. This PAA-Na-solution was successfully regenerated by membrane distillation. Dye-containing wastewater was simulated with a 50 ppm Orange-Acid-8-solution, which was the FS. Permeate flux in experiments without DS regeneration decreased from 25 to 15.5 L/(m^2^·h) within the 2 h experiment (Figure 21b).

Gu et al. investigated brown coal powder as DS to concentrate dye-containing textile wastewater [50]. Permeate fluxes were 0.979 and 0.900 L/(m^2^·h). The moistened brown coal after FO is supposed to be mixed further with water to create coal water slurry. This coal water slurry can then be used as a substitute for fossil fuel in gasification and chemical synthesis processes. Dye-containing wastewater would be concentrated facilitating further treatment. No DS regeneration is necessary in this application.

**Figure 22:**
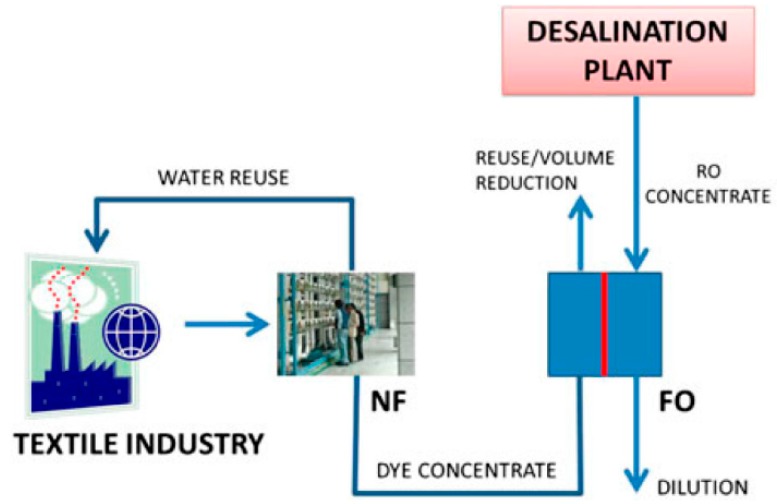
Proposed treatment scenario of dye-containing wastewater (reprinted from [147] with permission of Balaban Publishers—Desalination Publications).

Three different dye solutions were investigated as FS for forward osmosis in another study [147]. Real seawater was the DS. Permeate fluxes were between 1.62 and 3.47 L/(m^2^·h) depending on the dye concentration, membrane orientation and experiment duration. Dye rejection was almost 100%. FO performance was compared to NF that obtained permeate fluxes around 30 L/(m^2^·h) and dye rejection of more than 99%. As conclusion a textile wastewater treatment was proposed: (1) NF to treat textile wastewater; (2) FO with NF concentrate as FS and RO brine from seawater desalination as DS (Figure 22).

In another study, FO experiments were conducted with polayacrylamide (PAM) as DS [151]. Dye-containing wastewater was the FS. Permeate fluxes were between 2.65 and 5.14 L/(m^2^·h) depending on membrane orientation and experiment duration. Membrane fouling occurred with dye wastewater but was found out to be neglectable. Dye rejection was almost 100% regardless which membrane orientation was used. PAM was compared to KCl representing a conventional DS. Permeate water fluxes were more stable and reverse salt flux was lower with PAM. It was proposed that the diluted PAM solution could either be used in oil field extraction or be regenerated and recycled by UF, MD or heating processes.

### 3.8. Pulp and Paper Production

**Figure 23:**
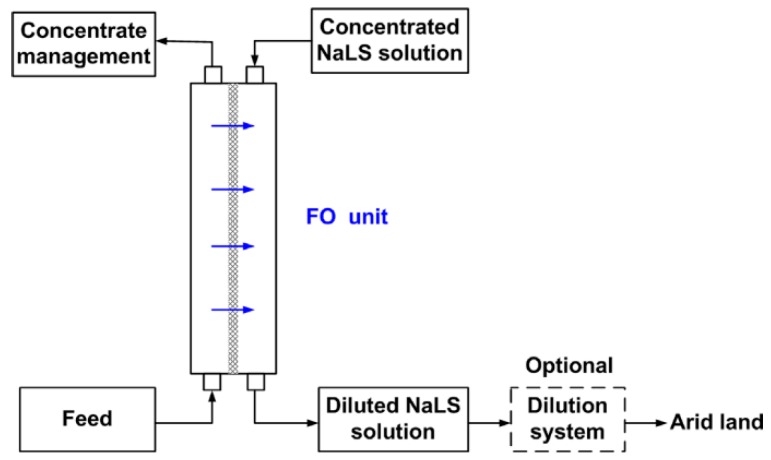
FO application scenario with usage of NaLS (waste product from pulp manufacturing) as DS (reprinted from [152] with permission from Elsevier).

To the authors’ knowledge, only little FO research is done regarding the pulp and paper industry. Duan et al. evaluated sodium lignin sulfonate (NaLS), a waste product from pulp production, as draw solution [152]. The diluted solution could be used for desert restoration to stabilize sand (Figure 23). Moreover, NaLS is a good substrate for plant growing.

In their experiments, Duan et al. used DI water and saline water as FS. As a result, permeate flux was 15 and 10 L/(m^2^·h) for the two membranes with deionized water as FS (600 g/kg NaLS solution as DS, ALDS membrane orientation). In this case, the osmotic pressure difference was 78 bar. The same DS combined with 30,000 mg/L NaCl as FS resulted in 5 and 2 L/(m^2^·h) permeate flux (ALFS membrane orientation). The lower permeate fluxes can be explained by the lower osmotic pressure difference. The FO application for NaLS dilution is similar to the FO application in fertigation. To use the NaLS solution for sand stabilization, a dilution down to 1–2% is necessary which is equivalent to an osmotic pressure of 1.3–2.7 bar. Only brakish water (2000 mg/L NaCl equal to an osmotic pressure of 1.5 bar) or less saline waters as FS could result in a NaLS solution that was directly applicable. If for example sea water (30,000 mg/L NaCl, π = 23 bar) is used as FS, the NaLS DS can be diluted down to 17% only. Thus, another dilution step would be necessary. Nevertheless, a promising FO application scenario is proposed.

### 3.9. Electronic Industry

In electronic industry, wastewater streams occur that contain valuable substances, e.g., heavy metals. However, these substances are often toxic or harmful and have to be removed from the wastewater. At the same time, this removal offers the chance to regain and recycle these substances back into the production process.

**Figure 24:**
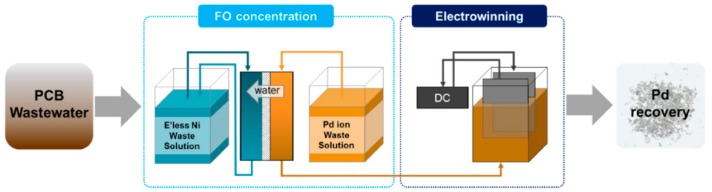
Forward osmosis application scenario at a PCB plant for palladium recovery (reprinted from [154] with permission from author).

Gwak et al. utilized forward osmosis to treat wastewater from a printed circuit board (PCB) manufacturing (Figure 24) [153]. Palladium containing wastewater as FS was concentrated up to 90%. This way, palladium could be regained efficiently by electrowinning. Nickel containing wastewater from electroless nickel plating was the DS. The diluted DS could be disposed to a wastewater treatment plant. No DS regeneration process is necessary in this case. Gwak et al. [154] mention that inorganic fouling occurred on the FS side and needed more investigation. However, they also say that in PCB manufacturing, other high conductivity waste streams are available. Further FO steps using these waste streams as DS could increase the palladium concentration even more.

Nguyen et al. describe lab-scale experiments in which they examined forward osmosis treatment for two wastewaters from a thin film transistor liquid crystal display (TFT-LCD) plant [155]. They used potassium iodide wastewater from the polarizer process as FS. Potassium hydroxide wastewater was applied as DS originating from the developing process. The iodide concentration in the FS increased from 0.6 to 6.9% during 120 h FO treatment enabling a recycling. Here, FO could replace conventional technologies like thermal distillation and reverse osmosis. The diluted DS could also be reused in the manufacturing process. Thus, a DS regeneration is not necessary.

### 3.10. Car Manufacturing Wastewater

Different wastewaters from car manufacturing industry were used for FO experiments [115]. The wastewaters were either used as FS or DS. If the tested wastewater was not combined with another wastewater, deionized water and 1 mol/L NaCl were used as FS and DS, respectively. Automobile cooling tower water and wastewater from cathodic dip painting were the tested DS. However, permeate fluxes were below 1.1 L/(m^2^·h). Rinsing water and wastewater from automobile cathodic dip painting as well as wastewater from automobile paint shop pre-treatment were the tested FS and showed good performance regarding the permeate flux between 7.5 and 19.4 L/(m^2^·h).

### 3.11. General Industrial Application

Several researchers who conducted FO experiment focused on the behaviour of single chemical elements during FO process, e.g., heavy metals [156,157]. Sometimes these studies examined also other points of interest like the performance of a newly developed FO membrane [49,158] or wastewater treatment [159,160]. In regard of the chosen elements, deposition on the membrane, diffusion through the membrane, and rejection by the membrane were investigated. Table 2 shows which heavy metals were studied.

The early FO research used a FO set-up without circulation and commercial RO membranes. Here, permeate flux did not exceed 4.5 L/(m^2^·h) and high diffusion rates for both DS substances and heavy metals were observed. In the recent studies, flat-sheet FO membranes, either commercial or self-manufactured, were applied and a circulation lab-scale set-up was used as described before [49,156,157,158,159,160]. Heavy metal rejection was high between 85% and 99.9%. Permeate fluxes ranged from 4 to 69 L/(m^2^·h).

To control process temperatures, many manufacturing industries use large amounts of cooling water, which are often recirculated in closed cooling loops. Due to evaporation losses, this cooling water is concentrated and has to be diluted with fresh water intermittently. This water is called make-up water. Furthermore, to remove substances from this cooling water loop, a certain amount of concentrated cooling water is discharged regularly.

Wang et al. investigated the usage of rainwater as make-up water [161]. They conducted FO experiments with rainwater as FS and cooling water from a steam plant as DS. This way, pure water was transported into the cooling water. The average flux was 1.75 L/(m^2^·h) at 23 °C. Increasing DS temperature from 3 to 50 °C lead to a 10 times higher FO permeate flux. Fouling did not show negative impact on the FO process.

Cooling tower water from automobile industry was the DS in other FO experiments [115]. Here, deionized water and wastewater from paintshop pre-treatment were the corresponding FS producing only low average permeate fluxes of 1.1 and 0.1 L/(m^2^·h), respectively.

## 4. Concluding Remarks

Forward osmosis is a promising solution for the energy-efficient water usage in manufacturing industries. In this paper, 51 original research articles were evaluated in which forward osmosis application in industries was investigated. So far, research was conducted on FO application in food and beverage industry, chemical industry, pharmaceutical industry, coal processing, micro algae cultivation, textile industry, pulp and paper industry, electronic industry, and car manufacturing. Articles were also published about heavy metal elimination and cooling water treatment; both of which might be related to industries. Forward osmosis was either applied for wastewater treatment, for the dilution of a fluid product or the concentration of a fluid product.

Unfortunately, it is not possible to compare the efficiency of the different application experiments due to varying experimental set-ups, operation conditions, and data interpretation. For this reason, it is also difficult to evaluate the potential of FO application in the industrial sectors. Certainly, one approach is the comparison of the obtained permeate fluxes: If the permeate flux is low, FO might not be a suitable treatment technology. However, the given permeate fluxes cannot be compared as they range from initial short-time permeate fluxes to long-time average permeate fluxes. Furthermore, the FO potential has to be evaluated individually for each application scenario because more aspects require consideration. These aspects are, for example, economic benefits, alternative treatment technologies, and legal requirements.

**Figure 25:**
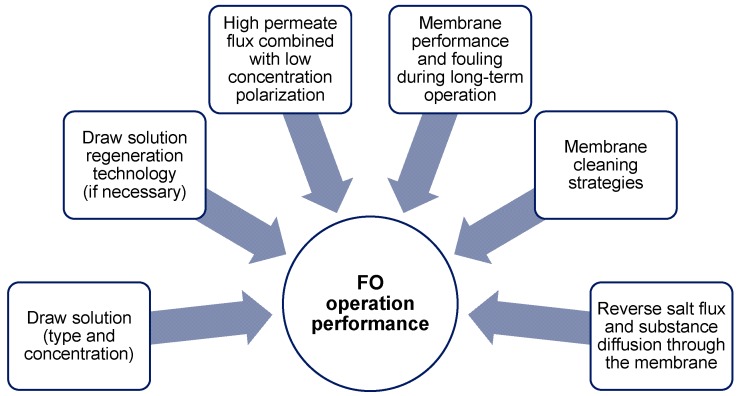
Crucial points for forward osmosis operation performance.

The general principle of forward osmosis is not questioned in the evaluated research papers. Full-scale implementation of forward osmosis in seawater desalination shows that forward osmosis is an applicable treatment technology. In regard to the industrial applications only basic proof-of-principle studies were conducted in lab-scale. The up-scaling on pilot- or full-scale will be the next step to optimize the operation and implement FO in industrial water and wastewater treatment. To further promote forward osmosis in industries, more research needs to be done. Crucial points are illustrated in Figure 25.

By conducting more research, it should be possible to establish forward osmosis as a treatment technology in manufacturing industries. It should also be possible to find more application scenarios. Advantageous would be the combined treatment of two streams where no synthetic draw solution would be necessary. So far, most research papers investigated FO application in one industry only. However, in order to benefit from the simultaneous treatment of two fluids in the forward osmosis process, water and wastewater streams in industrial parks with numerous industry branches should be investigated. Besides the technical applicability, energetic and economic benefits of forward osmosis need to be critically evaluated for each application scenario before full-scale implementation.

## Figures and Tables

**Table 1:** Parameters to be indicated with lab-scale forward osmosis experiments.

Feed Solution	Draw Solution	Membrane	Operation
type initial volume	type initial volume	type supplier active surface area orientation (ALFS/ALDS)	type of circulation (concurrent, countercurrent etc.) Flow velocity across membrane (or flow rate and flow channel dimensions) duration of experiment cleaning procedures

**Table 2:** Investigated heavy metals for FO treatment.

As	Cd	Cr	Cu	Hg	Ni	Pb	Zn	Ref.
	☑		☑			☑		[156]
	☑		☑		☑	☑	☑	[49]
	☑		☑			☑	☑	[158]
				☑				[160]
					☑			[159]
☑	☑		☑	☑		☑		[157]
		☑	☑					[42,43]

## Supplementary Materials

The following are available online at http://www.mdpi.com/2077-0375/8/3/47/s1, Table S1: Lab-scale set-ups used for investigation of forward osmosis application in manufacturing industries.
