# Supplementary material for: Forward Osmosis Application in Manufacturing Industries: A Short Review
Source: Membranes (Basel). 2018 Jul 23;8(3):47. doi: 10.3390/membranes8030047 (PMC6160976; doi:10.3390/membranes8030047)
Supplement: Supplementary file 1 [file membranes-08-00047-s001.pdf]

membranes

Table S 1. Lab-scale set-ups used for investigation of forward osmosis application in manufacturing industries

Branche of Industry	FS (type + volume)	DS (type + volume)	Membrane Area; Flow channel	Membrane type	Membrane Orientation	Flow Features	Temp.	Duration	Permeate Flux/Concentration factor/Yield	Hybrid technology	Lit.
**3.2 Food & Beverage Industry**											
Dairy (whey)	Whey protein powder + DI water (6%) 3 L	NaCl (0.3/0.5M/1.0); 8 L	106 cm^2 (15 fibers)	hollow fiber FO; self-manufactured; TFC	ALFS	circulation; counter-current; DS 22 cm/s; FS 55 or 15 cm/s	22.5±1.5°C (room temperature)	8 h 22 h	start permeate flux 10.3 and 11.7 L/(m^2·h); 10 % decrease	-	[112]
Dairy (whey)	Whey 3 L	NH_4HCO_3 (2 M); 3 L	140 cm^2	3 RO-membranes (Hydronautics); CTA (HTI)	ALFS ALDS	circulation; concurrent; 50 cm/s	30±0.5°C	4 h	start permeate flux 12 L/(m^2·h) end permeate flux 5 L/(m^2·h)	-	[110, 111]
Dairy (whey)	Acid cheese whey; 3.5 L	2 M NaCl (constant concentration); 3,5 L	140 cm^2	flat sheet FO; CTA, HTI (USA)	ALFS	circulation; concurrent; 50 cm/s	25±0.5°C (constant)	21 h (fresh FS 4 times)	Water recovery 66…68 %	MD	[107]
Dairy (whey)	Acid cheese whey; 3.5 L	2 M NaCl and 2 M NH_4HCO_3 (constant concentration); 3,5 L	140 cm^2	flat sheet FO; CTA (HTI)	ALFS	circulation; concurrent; 50 cm/s	25±0.5°C 30±0.5°C (constant)	14 h (fresh FS 3 times)	permeate volume 2.7 L and 1.6 L; start permeate flux 28.5 and 8,5 L/(m^2·h)	RO; thermolytic RO	[108]
Dairy (whey)	Acid cheese whey; 3 L	3 M NaCl; 3 L	140 cm^2	flat sheet FO; CTA (HTI)	ALFS	circulation; concurrent; 25/50/75/100 cm/s (150/300/450/600 L/h)	20/25/30/35/40 ±0.5°C (constant)	6 h	permeate volume 1.6 L, dry matter increased from 6.8 to 14.3 %	MF	[109]
Dairy (whey) Acetic acid production	1) whey (protein separation) 2) acetic acid	1 M $MgSO_4$	-	flat sheet, polyamide, NF-membrane	-	circulation; counter-current	-	-	permeate flux ca. 19 and 25 L/(m^2·h)	MF, UF, NF, fermentation	[113]
Dairy (wastewater)	Dairy wastewater (after 24 h precipitation); 2 L	1 M NaCl; 2 L	-	flat sheet; CTA-ES (HTI), AQP (Sterlitech)	ALFS	circulation; concurrent; 0.5 L/min	20±2 °C (room temperature)	3x6 h		MD	[114]

Branche of Industry	FS (type + volume)	DS (type + volume)	Membrane Area; Flow channel	Membrane type	Membrane Orientation	Flow Features	Temp.	Duration	Permeate Flux/Concentration factor/Yield	Hybrid technology	Lit.
Dairy (wastewater)	I) RO concentrate from dairy wastewater treatment plant; II) DI water; 1 L	III) 1 M NaCl; IV) cheese brine; 1 L	48 cm²; 1200x40x0.86 mm	flat-sheet; CTA (HTI)	ALFS	circulation; counter-current; 20 cm/s	room temperature	3x5 h	average permeate flux = 15.1 L/(m²·h) (I)+(IV) = 21.0 L/(m²·h) (II)+(IV) = 7.9 L/(m²·h) (II)+(III)	-	[115]
Food & Beverage (Tea extract)	tea extract; 2 L	5 M $CaCl_2$; 8 L	540 cm²	hollow-fiber membrane contactor (1.7 55 MiniModule, 3M Deutschland GmbH, Wuppertal, Germany)	tea extract shell side	circulation; counter-current	23±2°C	5 h	tea concentration of 40 %	-	[116]
Food & Beverage (Distillery)	sugarcane molasses distillery wastewater	3/4 M $MgCl_2.6H_2O$	43 cm²	flat-sheet FO; TFC (Aquaporin A/S)	ALFS	circulation; counter-current; 1 L/min	-	5x24 h	J_W = 2.8 L/(m²·h); water recovery (24 h) = 70%; melanoidin rejection 85-90%	-	[118]
Food & Beverage (Olive mill)	a) Olive mill wastewater (filtered); b) UF permeate of FO-concentrated olive mill wastewater; 3 L	$MgCl_2{\cdot}6H_2O$; 3.7 M (1,8-7,5 M); 4 L	200 cm²; 200x50x6 mm	flat sheet FO; CTA (HTI)	ALFS	circulation; concurrent; 6 cm/s (2.0-8.6 cm/s)	-	24 h (10 d)	a) J_W = 7.1-9.8 L/(m²·h); volume reduction 71%; b) J_W = 5 L/(m²·h); volume reduction 64%	filtration, MF, UF, NF	[117]
Food & Beverage (Grain processing); CO_2 sequestration	wastewater from grain processing	a) brine from CO_2 sequestration site; b) 20 % $MgSO_4$	42 cm²; Sterlitech CF042-FO	flat-sheet FO; (Aquaporin A/S)	ALDS	circulation; counter-current; 1 L/min	-	1-100 h	a) J_W = 5-15 L/(m²·h); a) J_W = 3-4.5 L/(m²·h)	MD; **direct FO application**	[119]
**3.3 Chemical Industry**											

Branche of Industry	FS (type + volume)	DS (type + volume)	Membrane Area; Flow channel	Membrane type	Membrane Orientation	Flow Features	Temp.	Duration	Permeate Flux/Concentration factor/Yield	Hybrid technology	Lit.
Chemical Industry	secondary effluent from industrial wastewater treatment plant (probably chemical industry), preconcentrated by UF or RO; -	NaCl, Na_2SO_4, $MgCl_2$ (1 M); *reference to other literature (Zhao et al. 2016): constant concentration*	*reference to other literature (Zhao et al. 2016): 33.15 cm^2; 8.5x3.9x 0.2cm*	flat sheet FO; (Toray Chemical Korea Inc.)	ALFS	circulation; counter-current; - (450 mL/min) *reference to other literature (Zhao et al. 2016): 12,9 cm/s*	25°C (constant)	*reference to other literature (Zhao et al. 2016): 11 h*	for 67% recovery: DS = $MgCl_2$: J_W = 13.0 L/(m^2·h) DS = Na_2SO_4: J_W = 8.08 L/(m^2·h) DS = NaCl: J_W = 9.63 L/(m^2·h)	FS pretreatment (concentrate further treated with FO): UF+RO+softening,	[57]
Chemical Industry	pretreated esterification wastewater		-	-					J_W declined within first 5 h from 9.56 to 6.0 L/(m^2·h)		[120]
Chemical Industry	PVC latex; 273 g	NaCl (0.3-1.8 M) = synthetic seawater; 1500 g	32 cm^2	flat-sheet FO; 2 CTA (HTI)	ALFS	circulation of DS only, FS stirred in tank above membrane; DS 1 L/min	20±2°C (room temperature)	24 h	start J_W = 8 and 4.5 L/(m^2·h); after 24 h PVC latex 75wt%	**direct FO application**	[48]
Chemical Industry (Ammonia absorption)	anaerobically digested sludge centrate from municipal WWTP	wastewater from ammonia absorption	42 cm^2; Sterlitech CF042-FO	flat-sheet FO; CTA-NW (HTI) + AIM (Aquaporin)	ALFS	circulation; counter-current; - (0.5 L/min)	-	72 h	end J_W = 3.56 and 3.13 L/(m^2·h) (AIM and CTA-NW)	**direct FO application**	[121]
Chemical Industry (Ammonia absorption)	activated sludge	wastewater from ammonia absorption	42 cm^2; Sterlitech CF042-FO	flat-sheet FO; CTA-NW (HTI)	ALFS	circulation; counter-current; - (0.5 L/min)	-	35 d	J_W = 1-3 L/(m^2·h)	FS pretreatment (bioreactor) = OMBR	[87]
Chemical Industry (Fermentation)	succinic acid (10/20/30/40 g/L); 1.0 L	NaCl (1/3/5 M), real seawater; 0.6/1.0 L	42 cm^2; Sterlitech CF042-FO	flat-sheet FO; CTA-ES (HTI)	ALFS	circulation; counter-current; -	25±1°C (room temperature)	2.5 h	J_W = 0-4.8 L/(m^2·h)	fermentation; **direct FO application**	[122]
Chemical Industry	acetic, butyric, valeric, and lactic acid (carboxylic acids, 10 mM); 1 L	NH_4Cl (1 M); 0.5 L	42 cm^2; 9.207x4.572x 0.23cm	flat-sheet FO; TFC-ES (HTI)	ALFS	circulation; counter-current; 25 cm/s	28±0.5°C (air conditioning)	30 h	J_W = 4.8 L/(m^2·h)	-	[129]

Branche of Industry	FS (type + volume)	DS (type + volume)	Membrane Area; Flow channel	Membrane type	Membrane Orientation	Flow Features	Temp.	Duration	Permeate Flux/Concentration factor/Yield	Hybrid technology	Lit.
Chemical Industry	lactic acid (8%); a) 2 L (2 kg) b) 3 L (3 kg)	glucose (60%); a) 0.7 L (1 kg) b) 2.8 L (4 kg)	a) 42 cm²; Sterlitech CF042P-FO; a) 140 cm²; Sterlitech SEPA CF-FO	flat-sheet FO; I) TFC (Aquaporin) II) TFC (Toray)	ALFS	circulation; concurrent; - (1-1.2 L/min)	20/40/60°C (constant)	a) 2h b) ca. 980 h	a) J_W = 2.1-10.0 L/(m²·h) (Aquaporin); J_W = 3.7-10.0 L/(m²·h) (Toray); b) J_W = 12 L/(m²·h) (Toray)	fermentation; **direct FO application**	[125]
Chemical Industry (Fermentation)	butyric acid (2 g/L); 1 L	$MgCl_2$ (5 M); 20 L	40 cm²; 4x10x0.5cm	flat-sheet FO CTA (HTI); flat-sheet RO TFC (XLE)	-	circulation; concurrent; 12.5 cm/s (1.5 L/min)	25°C	50% water recovery	J_W = 16-18 L/(m²·h)	fermentation; NF; RO	[124]
Chemical Industry (Fermentation)	a) crude glycerol (1/2/5%) b) pretreated and enzymatically hydrolysed wheat straw (PHWS) (5/20%)	a) crude glycerol (100%) b) pretreated and enzymatically hydrolysed wheat straw (PHWS) (100%)	33.15 cm²; 8.5x3.9x2.3cm; Sterlitech Acrylic CF042A-FO	flat-sheet FO; Aquaporin	ALFS	circulation; counter-current; a) 0.1 cm/s (50 mL/min); b) 1.7 cm/s (173 mL/min)	37°C (constant)	15 h	a) J_W = 8.4, 9.0, 10.5 L/(m²·h) (5/2/1%); b) J_W = 1.3, 5.4, 6.2 L/(m²·h) (20/5/0%)	fermentation; **direct FO application**	[126]
Chemical Industry (Fermentation)	a) model sugar solution (20 g/L xylose + 0.45 g/L acetic acid) b) liquid fraction from rice straw pretreated with hot water	I) 2.5 M NaCl; switchable polarity solvents (SPS): II) 3.6 M Triethylamine-carbon dioxide (TEA-CO2) ; III) 4.3 M Trimethylamine-carbon dioxide (TMA-CO2) (constant concentration)	-	flat-sheet FO; TFC-ES (HTI)	ALFS	circulation; counter-current; - (FS 0.4 L/min; DS 0.75 L/min)	-	a) 48 h; b) 72 h	a) start J_W = 8.8, 5.7, 2.9 L/(m²·h) (I/II/III); after 48 h J_W < 0.8 L/(m²·h); b) start J_W = 4 L/(m²·h) (II); after 48 h J_W = 1.8 L/(m²·h)	fermentation; **direct FO application**	[127]
Chemical Industry (Fermentation)	liquid fraction from rice straw pretreated with hot water	switchable polarity solvent (SPS): 3.6 M Triethylamine-carbon dioxide (TEA-CO2)	43 cm²	flat-sheet FO; TFC-ES (HTI)	ALFS	circulation; counter-current; - (FS 0.4 L/min; DS 0.75 L/min)	-	72 h	start J_W = 3.9 L/(m²·h); after 48 h J_W = 1.8 L/(m²·h); total sugar content produced 107 g/L;	fermentation; NF; enzymatic hydrolysis; **direct FO application**	[128]
**3.5 Pharmaceutical Industry**											

**Branche of Industry**	**FS (type + volume)**	**DS (type + volume)**	**Membrane Area; Flow channel**	**Membrane type**	**Membrane Orientation**	**Flow Features**	**Temp.**	**Duration**	**Permeate Flux/Concentration factor/Yield**	**Hybrid technology**	**Lit.**
Pharmaceutical Industry	I) tetracycline (0, 1000, 2000, 5000, 10000 ppm) dissolved in ethanol<br>II) tetracycline (0, 2000 ppm) in isopropanol (IPA)<br>III) triglycerides (0, 20 wt%) dissolved in hexane	IV) LiCl (2 M)<br>V) 50 wt% methyl palmitate<br>VI) citric acid (2 M)<br>VII) polyethylene glycol (100 G/L PEG1000)<br>VIII) diethanolamine (2 M)	4 cm²	flat-sheet FO; TFC; self-manufactured	ALFS ALDS	circulation; counter-current; - (0.2 L/min)		2 h	average permeate flux ALDS & ALFS (no tetracycline/triglycerides)<br>(I+IV) = 3.82±0.37 & 2.52±0.14 L/(m²·h)<br>(II+IV) = 0.51±0.11 & 0.34±0.06 L/(m²·h)<br>(III+V)= 2.04±0.34 & 1.87±0.13 L/(m²·h)<br>(I+VI) = 2.85±0.72 & 2.05±0.30 L/(m²·h)<br>(I+VII) = 1.23±0.19 & 0.89±0.24 L/(m²·h)<br>(II+VIII) = 0.52±0.05 & 0.32±0.07 L/(m²·h)	-	[130]
Pharmaceuticak Industry	protein solution (bovine serum albumin = BSA solution); 0.5 L	NaCl; 0.5 L	-	hollow-fiber NF	ALFS	circulation; concurrent; 6 cm/s	22.5°C	2 h	Initial water flux (0.5/1/1.5/2 M NaCl) = 2.7/3.6/5.1/5.3 L/(m²·h)	MD	[161]
**3.5 Coal Processing**											
Coal processing	Coke-oven wastewater; 15 L	NaCl, $MgSO_4$, and $CaCl_2{\cdot}H_2O$; 15 L	150 cm²	flat sheet NF/MF; TFC (Sepro Membranes, USA)	ALFS	circulation; counter-current; 21-42 cm/s	consta nt	12 h/d for 7 days	42-46 L/(m²·h)	NF	[131]
Coal processing	3 Artificial coal gasification wastewaters (100 mg/L three different phenolic compounds); 2 L	NaCl (1.75-10.5%, concentration adjusted hourly); 2 L	60 cm²; 100x60x2 mm	flat sheet FO; TFC (HTI)	ALFS	circulation; concurrent; 14 cm/s (1 L/min)	FS 20°C, DS 30°C (consta nt)	4 h	8.5-10.5 L/(m²·h).	-	[133]
Coal processing	phenol solution; -	NaCl (0.5-4 M)	24 cm²; 80x30x2 mm	flat sheet FO; CTA & TFC (FTS) + self-manufactured	ALFS ALDS	circulation; concurrent; 25 cm/s	25±1°C (consta nt)	100 mL permeate	3-34 L/(m²·h)	-	[74]
**3.6 Micro Algae Cultivation**											

**Branche of Industry**	**FS (type + volume)**	**DS (type + volume)**	**Membrane Area; Flow channel**	**Membrane type**	**Membrane Orientation**	**Flow Features**	**Temp.**	**Duration**	**Permeate Flux/Concentration factor/Yield**	**Hybrid technology**	**Lit.**
Microalgae Cultivation	0.2 g/L algal suspension (3 different species); 1 L	sea salt solution (70 g/L); $MgCl_2$ (86.5 g/L); $CaCl_2$ (114.3 g/L); 6 L	200 cm²	flat-sheet FO; CTA (HTI)	ALFS	circulation; counter-current; 9.6 cm/s	25±1°C (constant)	until 75% permeate	start J_W = 7.0 L/(m²·h); final ΔJ_W = 5.3-70.9%; algae dewatering efficiency = 59-80%	microalgae cultivation	[139]
Microalgae Cultivation	0.2 g/L algal suspension (1 species); 1 L	sea salt solution (70 g/L); $MgCl_2$ (86.55 g/L); $CaCl_2$ (68.96 g/L); NaCl (68.96 g/L); 6 L	200 cm²	flat-sheet FO; CTA + TFC (HTI)	ALFS ALDS	circulation; counter-current; 9.6 cm/s	25±1°C (constant)	until 75% permeate	start J_W = 6.7-8.2 L/(m²·h); final JW = 1.5-5.9 L/(m²·h); final ΔJ_W = 10-59%;	microalgae cultivation	[140]
Microalgae (Cultivation)	0.1 g/L algal suspension (in 10 mM NaCl or 7 mM NaCl + 1 mM $MgCl_2$)	0.2-5 M NaCl; 0.15-1.5 M $MgCl_2$ (concentration raised stepwise every 30 min)	29.2 cm²	flat-sheet FO; CTA (HTI)	ALFS ALDS	circulation; concurrent	-	2-3 h		-	[141]
Microalgae (Cultivation)	0.1 g/L algal suspension (in 10 mM NaCl); 4 L	a) 0.3-5 M NaCl; b) 0.5/2.0 M $MgCl_2$; 5 L	60 cm²	flat-sheet FO; CTA (HTI)	ALFS ALDS	circulation; concurrent; 22.5 cm/s	22±1°C	4 h	a) J_W = 7-30 and 10-50 L/(m²·h) (ALFS and ALDS); b) J_W = 14-55 (ALDS);	-	[142]
Microalgae Cultivation	algae (0.5-2 g/L) in artificial medium or municipal wastewater	artificial or natural seawater	900 cm²	modified X-Pack Hydration Bags (HTI)	ALFS	batch; no circulation; membrane bag in water bath or ocean	-	4 h - 52 d	J_W = 2 L/(m²·h)	microalgae cultivation	[143]
**3.7 Textile Industry**											
Textile Industry	artificial dye-containing wastewater; 0.5 L	NaCl (1-2 M, constant)	10 cm²	flat-sheet FO; TFC, self-manufactured	ALFS ALDS	circulation; counter-current; 1.7 cm/s	22±0.5°C (room temperature)		start permeate flux 36 L/(m²·h), stable long-term permeate flux 12 L/(m²·h); dye rejection 99.9%	coagulation & flocculation	[147]
Textile Industry	artificial dye-containing wastewater (50 ppm Congo red); -	0.25 g/mL P(SSA-co-MA)-Na-1 (polyelectrolyte salt-poly sodium)	18.9 cm²	flat-sheet FO; TFC (HTI)	ALFS	circulation; -; - (300 mL/min)	25±1°C (room temperature)	2 h	3 L/(m²·h)	NF	[148]

Branche of Industry	FS (type + volume)	DS (type + volume)	Membrane Area; Flow channel	Membrane type	Membrane Orientation	Flow Features	Temp.	Duration	Permeate Flux/Concentration factor/Yield	Hybrid technology	Lit.
Textile Industry	artificial dye-containing wastewater (50 ppm Acid Orange 8); 500 mL	0.24/0.36/0.48/0.60 g/mL PAA-Na (poly acrylic acid sodium salt); 300 mL	-	hollow fiber FO; CTA, self-manufactured	ALDS	circulation; concurrent; - (FS 100 mL/min, DS 500 mL/min)	50±0.5° C, 60±0.5° C, 70±0.5° C, 80±0.5° C	2 h	permeate flux decreased from 25 to 15.5 L/(m²·h)	MD	[149]
Textile Industry; Coal gasification	artificial dye-containing wastewater (80 ppm methylene blue); 50 mL	brown coal powder; 1 g placed on membrane support layer	1.33 cm²	flat-sheet FO; CTA (HTI)	ALFS	no circulation!	room temperature	8-11 h	0.979 and 0.900 L/(m²·h) (first hour; two different brown coals)	**direct FO application**	[50]
Textile Industry	artificial dye-containing wastewater (15 g/L NaCl + 25/500/750/1000 mg/L of Acid Green 25 dye or Remazol Brilliant Orange or Remazol Blue); -	real seawater	71 cm²	flat-sheet FO; CTA (HTI)	ALFS ALDS	circulation; counter-current; - (1.5 mL/min)	23±1°C	2 h; 6 h	Average fluxes (2 h, ALFS) = 1,62…2,25 L/(m²·h) (250…1000 mg/L dye) Average fluxes (2 h, ALDS) = 2,12…3,47 L/(m²·h) (250…1000 mg/L dye) Average fluxes (6 h, ALFS) = 1,94…2,76 L/(m²·h) (1000 mg/L dye)	**direct FO application**	[146]
Textile Industry; Oil Industry	artificial dye-containing wastewater (0,05 g/L Reactive Brilliant Red K-2BP); 1000 mL	Polyacrylamide; 500 mL	20 cm²; 7.7x2.6x0.3 cm	flat-sheet FO; TFC	ALFS ALDS	circulation; concurrent; 10 cm/s	25±0.5° C	5 h	ALFS 3.21-2.65 L/(m²·h); ALDS 5.14-4.19 L/(m²·h)	**direct FO application**	[150]
**3.8 Pulp and Paper Production**											
Pulp production; desert restoration	DI water; saline feed (100/2000/30'000 mg/L NaCl)	sodium ligning sulfonate (NaLS; 600 g/kg)	50 cm²; 50x100x2.8 mm	flat sheet FO; 2 CTA (HTI)	ALFS ALDS	circulation; concurrent; FS 29 cm/s; DS 10 cm/s	23°C (constant)	-	FS=DI+ALDS: permeate flux 15 and 10 L/(m²·h); FS=30'000 mg/L NaCl+ALFS: permeate flux 5 and 2 L/(m²·h)	**direct FO application**	[151]
**3.9 Electronic Industry**											

**Branche of Industry**	**FS (type + volume)**	**DS (type + volume)**	**Membrane Area; Flow channel**	**Membrane type**	**Membrane Orientation**	**Flow Features**	**Temp.**	**Duration**	**Permeate Flux/Concentration factor/Yield**	**Hybrid technology**	**Lit.**
Electronic Industry (TFT-LCD plant)	KI wastewater from polarizer process (0.6% iodide); 1 L	KOH wastewater; 2 L	41.4 cm^2; 45x92x2 mm; Sterlitech	flat sheet FO; CTA & TFC (HTI)	ALFS ALDS	circulation; counter-current; 9.26 cm/s (500 mL/min)	25±0.5° C (constant)	92 h 120 h	average permeate (92 h) flux ALFS 4.9 L/(m^2·h) and ALDS 5.7 L/(m^2·h); iodide concentration in FS increased to 6.9% (120 h)	**direct FO application**	[154]
Electronic Industry (PCB plant)	Pd catalyst waste solution	electroless nickel plating solution	-	-	ALFS ALDS	circulation; concurrent	-	-	FS concentration yield > 90%; ALDS: J_W = 39,4 L/(m^2·h); $J_{S,Ni}$ = 0,43 g/(m^2·h) ALFS: ca. J_W = 19 L/(m^2·h); $J_{S,Ni}$ = 0,4 g/(m^2·h)	**direct FO application**	[153]
**3.10 Car manufacturing wastewater**											
Car Manufacturing	I) rinsing water from cathodic dip painting; II) wastewater from cathodic dip painting; III) wastewater from paint shop pre-treatment; IV) DI water; 1 L	V) cooling tower water; VI) wastewater from cathodic dip painting; VII) 1 M NaCl; 1 L	48 cm^2; 1200x40x0.86 mm	flat-sheet; CTA (HTI)	ALFS	circulation; counter-current; 20 cm/s	room temperature	3x5 h	average permeate flux = 12.1 L/(m^2·h) (I)+(VII) = 1.1 L/(m^2·h) (IV)+(V) = 7.5 L/(m^2·h) (III)+(VII) = 0.3 L/(m^2·h) (VI)+(VI) = 19.4 L/(m^2·h) (II)+(VII) = 0.1 L/(m^2·h) (III)+(V)	-	[115]
**3.11 General industrial application**											
Heavy Metal Elimination	2 g/L $CuSO_4/Pb(NO_3)_2/CdCl_2$	2 M NaCl	9 cm^2	flat-sheet FO; TFC, self-manufactured	ALFS	probably no circulation; batch	-	-	J_W = 45-50 L/(m^2·h); heavy metal rejection > 99.4%	-	[155]
Heavy Metal Elimination	1/2/5 g/L Cu^{2+}, Ni^{2+}, Pb^{2+}, Zn^{2+}, Cd^{2+}	0.5/1.0/1.5/2.0 M $MgCl_2$	9 cm^2	flat-sheet FO; self-manufactured	ALFS ALDS	no circulation; batch	25/45/65°C	6 h	AL-DS: rejection > 95.93 %; 23,5 L/(m^2·h); AL-FS: rejection > 99.32 %; 14 L/(m^2·h) (DS = 1 M MgCl2; FS = 2 g/L metal solution)	-	[49]
Heavy Metal Elimination	0.05-1 g/L Cd^{2+}, Pb^{2+}, Cu^{2+}, Zn^{2+} (pH 4.5±0.5)	0.5-2 M NaCl;	-	flat-sheet FO; self-manufactured	-	circulation; concurrent; - (260 mL/min)	25°C (constant)	-	J_W = 27.3-69 L/(m^2·h) (DS = 0.5-12.0 M); heavy metal rejection = 94-85% (0.2-1 g/L FS)	-	[157]
Heavy Metal Elimination	0.02-1 mg/L $HgCl_2$; 1 L	0.5-2 M NaCl; 0.5-2 M $MgCl_2$; 1 L	42 cm^2; Sterlitech CF042-FO; 9.2x4.6x0.2cm	flat-sheet FO; TFC (HTI)	ALFS	circulation; counter-current	-	-	J_W = 4-9.5 L/(m^2·h); mercury rejection = 98%	-	[159]

**Branche of Industry**	**FS (type + volume)**	**DS (type + volume)**	**Membrane Area; Flow channel**	**Membrane type**	**Membrane Orientation**	**Flow Features**	**Temp.**	**Duration**	**Permeate Flux/Concentration factor/Yield**	**Hybrid technology**	**Lit.**
Heavy Metal Elimination	0.1 g/L $NiCl_2$ + 0/50/100 mg/L NaCl (+ detergent); 1 L	117/155/194.5 mg/L NaCl; 1 L	20 cm^2; 7.7x2.6x0.3cm	flat-sheet FO; CTA & TFC (HTI)	ALFS ALDS	circulation; concurrent; 10 cm/s	25°C (constant)	5 h	J_W = 5-34 L/(m^2·h);	-	[158]
Heavy Metal Elimination	1/2/5 g/L $Cr_2O_7^{2+}$, $HAsO_4^{2-}$, Pb^{2+}, Cd^{2+}, Cu^{2+}, Hg^{2+}; 0.4 L	bulky hydroacidcomplex Na4[Co(C6H4O7)2] · 2H2O (Na–Co–CA) (1,0/1,5 M); 0.1 L	4 cm^2	flat-sheet FO; TFC, self-manufactured	ALFS	circulation; concurrent	23/40/50/60°C	0.5 h	J_W = 10-17 L/(m^2·h); heavy metal rejection > 99.7%	-	[156]
Heavy Metal Elimination; Food Industry	Copper solution; chromium solution; wastewater from fish and shell fish processig plant	synthetic seawater; concentrated sugar solution	13 cm^2; 58 cm^2	flat-sheet RO; 10 commercial membranes	ALFS ALDS	no circulation; concurrent	room temperature	< 6 h	J_W < 4.5 L/(m^2·h)		[42,43]
Cooling Water	rainwater	cooling water from a steam plant	20 cm^2	flat-sheet FO; CTA (HTI)	ALFS	circulation; counter-current; 45/100 mL/min	3-50 °C	1.5 h	average J_W (23°C) = 1.75 L/(m^2·h);		[160]
Cooling Water	I) wastewater from automobile paint shop pre-treatment; II) DI water; 1 L	III) cooling tower water; 1 L	48 cm^2; 1200x40x0.86 mm	flat-sheet; CTA (HTI)	ALFS	circulation; counter-current; 20 cm/s	room temperature	3x5 h	average permeate flux = 1.1 L/(m^2·h) (II)+(III) = 0.1 L/(m^2·h) (I)+(III)	-	[115]
